# Correlates of physical activity behaviour in a population sample with increased cardiovascular risk and established cardiovascular disease: Cross-sectional analysis of data from the Paracelsus 10,000 prospective cohort study in Salzburg, Austria

**DOI:** 10.1371/journal.pone.0356607

**Published:** 2026-08-19

**Authors:** Stefan T. Kulnik, Anna E. Carrozzo, Devender Kumar, Melanie Roth, Jens Blechert, Rik Crutzen, Patrick Langthaler, Martin Pühringer, Ludmilla Kedenko, Arne C. Bathke, Bernhard Iglseder, Bernhard Paulweber, Eugen Trinka

**Affiliations:** 1 Salzburg Research Forschungsgesellschaft mbH, Salzburg, Austria; 2 Ludwig Boltzmann Institute for Digital Health and Prevention, Salzburg, Austria; 3 Faculty of Engineering, University of Southern Denmark, Odense, Denmark; 4 Department Health Sciences, Salzburg University of Applied Sciences, Puch/Salzburg, Austria; 5 Department of Psychology, Paris Lodron University of Salzburg, Salzburg, Austria; 6 Department of Health Promotion, Maastricht University, Maastricht, Netherlands; 7 Department of Neurology, Christian Doppler University Hospital, Paracelsus Medical Private University and Centre for Cognitive Neuroscience, Member of the European Reference Network EpiCARE, Salzburg, Austria; 8 Department of Sport and Exercise Science, Paris Lodron University of Salzburg, Salzburg, Austria; 9 Department of Internal Medicine I, University Hospital Salzburg (SALK), Paracelsus Medical Private University, Salzburg, Austria; 10 Department of Artificial Intelligence and Human Interfaces, Paris Lodron University of Salzburg, Salzburg, Austria; 11 Team Biostatistics and Big Medical Data, IDA Lab Salzburg, Paracelsus Medical Private University, Salzburg, Austria; 12 Department of Statistics, University of Kentucky, Lexington, Kentucky, United States of America; 13 Department of Geriatric Medicine, Christian Doppler University Hospital, Paracelsus Medical Private University, Salzburg, Austria; 14 Department of Public Health, Health Services Research and Health Technology Assessment, UMIT – University for Health Sciences, Medical Informatics and Technology, Hall in Tirol, Austria; 15 Neuroscience Institute, Christian Doppler University Hospital, Paracelsus Medical Private University and Centre for Cognitive Neuroscience, Salzburg, Austria; Kerman University of Medical Sciences, IRAN, ISLAMIC REPUBLIC OF

## Abstract

Regular physical activity (PA) remains challenging for many people with high cardiovascular risk and established cardiovascular disease (CVD). We aimed to study correlates of PA behaviour in a population sample with increased cardiovascular risk and established CVD in Austria. We analysed cross-sectional data from the Paracelsus 10,000 study, a population-based cohort study of 40–70-year-olds in the city of Salzburg, Austria, and its surroundings. In a sub-sample of participants with increased cardiovascular risk and established CVD, we examined the associations between 29 correlates and leisure-time PA (weekly hours of walking, cycling and doing sports) as the primary PA outcome, as well as four secondary PA outcomes. We used stepwise multiple regression and machine learning models (decision trees, support vector machines, k-nearest neighbours, random forest and gradient boosting) to examine these associations. We analysed data from 1,173 participants with mean age 60.5 years (range 42–72 years). Nineteen percent of participants were female. Across the different PA outcomes and statistical analysis models, retirement, more PA during earlier life decades, and adherence to a Mediterranean diet were positively associated with PA behaviour. Greater comorbidity and depression were inversely associated with PA. In stepwise multiple regression, adjusted effect estimates on the primary PA outcome were 1.5 (SE 0.7, p = 0.0310) for retirement, 4.1 (SE 3.1, p < 0.0001) for past PA behaviour, 0.5 (SE 0.2, p = 0.0027) for Mediterranean diet, and −0.2 (SE 0.1, p = 0.0180) for depression. These findings contribute to a better understanding of the correlates of PA behaviour in populations at increased cardiovascular risk and with established CVD and may inform approaches for selective and personalised PA promotion strategies in cardiovascular care.

## Introduction

Cardiovascular diseases (CVDs) are the leading cause of death, with an estimated 18.6 million deaths worldwide attributed to CVDs in 2019 [1]. CVDs include coronary heart disease, cerebrovascular disease, peripheral artery disease and other conditions [2]. Lack of physical activity is a critical contributing risk factor for CVDs, and regular physical activity constitutes a crucial component in the primary and secondary prevention of CVDs [3]. Despite the promotion of physical activity in both the primary and secondary prevention of CVDs, including structured and supervised cardiac rehabilitation programmes, physical activity levels remain low [4]. Therefore, it is crucial to understand critical factors that are related to and might influence physical activity behaviour, particularly in groups with increased cardiovascular risk and established diagnoses of CVD. This understanding is a crucial first step towards accounting for these factors in the development and delivery of tailored physical activity promotion strategies [5,6]. Large scale cohort studies are needed to identify and accurately estimate the associations between such factors and physical activity.

Paracelsus 10,000 (P10) is a prospective cohort study conducted in the city of Salzburg, Austria, and its surroundings [7]. Between 2013 and 2020, the study recruited a randomly selected population-based sample of 10,044 participants (5,176 women) in the age group from 40 to 70 years. The focus of P10 lies on the systematic monitoring of common non-communicable diseases and their risk factors. To this end, a large set of cross-sectional data was collected from participants, including medical history, medical examinations, biological samples, and various survey instruments [7]. This rich dataset offers an opportunity to investigate multitudes of research questions, including aspects related to exercise physiology and physical activity [8–10].

Factors that are related to and might influence physical activity behaviour in the general adult population have been well-studied and include health status, past physical activity behaviour, self-efficacy, intention to exercise, age, sex, education level, ethnic origin, body weight, social support, aspects of the physical environment and others [6,11–13]. However, these factors might differ in individuals with increased cardiovascular risk or established CVD [5]. Several systematic reviews have focused specifically on this population, yet data from the Austrian context is lacking in this literature [14]. The P10 dataset offers an opportunity to explore this research question in a cardiovascular population sample local to Austria. We therefore conducted an analysis among P10 study participants with increased cardiovascular risk (primary prevention sub-group) and with an established diagnosis of CVD (secondary prevention sub-group). We aimed to describe the associations between participant characteristics (including socio-demographic, clinical, psychological/cognitive, lifestyle, and environmental factors) as independent variables and physical activity behaviour as dependent variable, and to examine how these participant characteristics relate to physical activity behaviour. Insight into such factors can inform the tailoring and personalisation of physical activity programmes in the primary and secondary prevention of CVD [6].

## Materials and methods

Details of the P10 study design have been published elsewhere [7]. In brief, 60,000 people aged 40–70 years and living in the city of Salzburg and its surroundings were randomly identified from the population register and invited by letter to participate in the study. Recruitment was stratified, aiming to include an equal number of men and women and 25%, 50% and 25% of participants in age groups 40–49 years, 50–59 years, and 60–70 years, respectively. Recruitment took place from 01 April 2013 until 31 March 2020, at which point 10,044 participants had been included in the study. Each study participant completed data collection in a single study visit on site at the University Hospital Salzburg in the presence of trained study personnel. Medical history, family history and medication were assessed by audio-recorded face-to-face interview. Study personnel specifically questioned participants whether each reported medical condition had been formally diagnosed by a medical doctor. All self-report questionnaires were completed by participants on a computer in the presence of study personnel who assisted as needed. Since autumn 2020, all study participants have been invited to return for their follow-up visit seven years after their initial study visit [7]. While the P10 study is a prospective cohort study, the data analysed for this article represents a cross-sectional dataset collected at the initial study visit.

The study received ethical approval from the research ethics committee of the County of Salzburg (reference 415-E/1521/6–2012). All study participants provided written informed consent.

For this analysis, we developed a prospective statistical analysis plan [15] and prospectively registered the analysis on the Open Science Framework (OSF) platform [16]. The purpose of this was to *a priori* define the research questions and corresponding statistical procedures, to increase methodological rigour and minimise the risk of selective reporting [17]. The reporting of this study follows the Strengthening the Reporting of Observational Studies in Epidemiology (STROBE) statement [18].

## Study population

We included P10 study participants with increased cardiovascular risk (primary prevention sub-group) and with an established diagnosis of CVD (secondary prevention sub-group). The primary prevention sub-group was selected according to the SCORE2 (for age groups 40–69 years) and SCORE2-OP (for age groups 70 + years) CVD risk categorisation, which describes the 10-year risk of suffering a fatal (death due to coronary heart disease, heart failure and sudden death) or non-fatal cardiovascular event (non-fatal myocardial infarction and non-fatal stroke) [3,19,20]. The SCORE2 and SCORE2-OP risk models are based on country of residence, age, sex, systolic blood pressure, blood cholesterol (high density lipoprotein and total cholesterol) and current smoking [19,20]. For the Austrian geographic region, the high risk SCORE2/SCORE2-OP category (orange) indicates risks of 2.5% to <7.5%, 5% to <10% and 7.5% to <15% for the age groups <50 years, 50–69 years and 70 + years, respectively. The very high risk SCORE2/SCORE2-OP category (red) indicates risks of ≥7.5%, ≥ 10% and ≥15% for the age groups <50 years, 50–69 years and 70 + years, respectively [3,19,20]. All P10 study participants without established diagnosis of CVD who were in the high or very high risk SCORE2/SCORE2-OP categories were included in the primary prevention sub-group.

For the secondary prevention sub-group we included all P10 study participants who indicated one or more of the following diagnoses in the interviewer-administered medical history questionnaire (ICD-10 codes [21] in brackets): coronary heart disease (I25.1), chronic heart failure (I50), peripheral arterial disease (I73.9), abdominal aortic aneurysm (I73.1, I73.4), atrial fibrillation (I48) and stroke (I64). Out of altogether 10,044 study participants in the P10 dataset, there were 3,625 in the primary prevention sub-group and 612 in the secondary prevention sub-group.

### Variables

#### Terminology.

In accordance with Bauman et al. [22], we acknowledge that our analysis of variables that might be related to and might influence physical activity behaviour is guided by an underlying logic of causal relationships; but that such conclusions cannot be drawn from cross-sectional observational data. We therefore use the term “correlates” to describe the variables under investigation, emphasising that these analyses present statistical associations/correlations, and not necessarily evidence of causality.

#### Correlates of physical activity behaviour.

From the variables available within the P10 dataset, we selected 29 to be examined as correlates of physical activity behaviour. These variables were selected because of their likely or potential relationship with individuals’ physical activity behaviour, based on published empirical research or theory [6,11–13,23]. Included were socio-demographic variables (age, sex, education level, occupation, migration background, religion, household income, marital status, number of children and retirement), clinical variables (CVD risk, comorbidity, body mass index, knee pain, hip pain and low back pain), psychological and cognitive variables (depression, daytime sleepiness and impulsivity), lifestyle variables (smoking status, alcohol use, past physical activity behaviour and Mediterranean diet) and environmental variables (number of people in the household, type of housing, size of living quarters, population density, environmental noise and air pollution).

Socio-demographic data were collected using a self-report questionnaire. CVD risk was defined as described above according to the SCORE2/SCORE2-OP risk categories and established diagnosis of CVD. In order from least to most severe CVD risk, the categories are: primary prevention sub-group with high risk, primary prevention sub-group with very high risk, and secondary prevention sub-group. Comorbidity was categorised in 0, 1–2 or ≥3 of the following diagnoses: type 2 diabetes, type 1 diabetes, coronary heart disease, chronic heart failure, peripheral arterial disease, rheumatoid arthritis, cirrhosis of the liver, chronic hepatitis, gastric or duodenal ulcer, chronic obstructive pulmonary disease, stroke, Alzheimer’s disease or other type of dementia, chronic kidney failure, pancreatic cancer, bladder cancer, lung cancer, breast cancer, brain tumour and ovarian cancer. Body mass index was calculated from clinician-measured anthropometric measurements (weight, height). Knee, hip and low back pain were measured using the Western Ontario and McMaster Universities Osteoarthritis Index (WOMAC) [24] and the Oswestry Disability Index (ODI) [25]. Depression, daytime sleepiness and impulsivity were measured with the Beck Depression Inventory (BDI) [26], the Epworth Sleepiness Scale (ESS) [27] and the Barratt Impulsiveness Scale (BIS-15) [28,29]. Smoking status was recorded from self-report. Alcohol use was measured using the Alcohol Use Disorder Identification Test (AUDIT) [30]. Past physical activity behaviour was recorded from self-reported average hours per week of doing sports at ages 5–19, 20–34, 35–49, and 50 + years. Adherence to a Mediterranean diet was measured using the Mediterranean Diet Adherence Screener (MEDAS) [31]. These standard questionnaires are considered valid and reliable for the general adult population. Environmental data were collected using a self-report questionnaire, including one Likert scale question each on environmental noise (street, railway and air traffic, industrial noise, neighbours) and air pollution (street and air traffic, residential burners, industrial pollution).

#### Outcome variables.

The outcome of interest was physical activity behaviour, measured by self-report using a questionnaire adapted from the European Prospective Investigation into Cancer and Nutrition (EPIC) programme [32]. This questionnaire has been validated in large European cohorts [32,33] and captures not only hours per week spent walking, cycling and doing sports, but also gardening, doing handiwork/repairs and/or housework in the past year. Of note, physical activity intensity was not captured, i.e., these data represent the sum of physical activity at light, moderate and vigorous intensity levels. From this, we summed hours per week spent walking, cycling and doing sports as our primary outcome variable, based on the predictive value of leisure-time physical activity on health outcomes [34]. Additionally, we explored the secondary outcome variables Cambridge Activity Index (CAI), derived from hours per week spent cycling and doing sports plus occupational physical activity, and Recreational Activity Index (RAI), derived from hours per week spent walking, cycling and doing sports. Both indices are ordinal variables with four activity levels (active, moderately active, moderately inactive, inactive) [33]. We further explored a physical activity outcome composed of hours per week spent gardening, doing handiworks/repairs and/or housework, which represent chores and maintenance-type physical activities in and around the home, and total physical activity (hours per week of all six physical activity behaviours).

### Data cleaning

Statistical analysis included only those study participants with complete datasets for all correlates and outcome variables described above. This resulted in a sample of 1,173 participants (1,018 in the primary prevention sub-group and 155 in the secondary prevention sub-group). We decided not to impute missing data because of complex patterns of missingness, and imputing a large number of correlates in this exploratory context was unlikely to provide clear statistical advantages. Moreover, imputation could also have introduced artefacts affecting the association estimates and the descriptive summaries of the analytic sample.

### Statistical analysis

The statistical analysis approach included bivariate analysis, interpretable parametric regression models (i.e., multiple linear regression, ordinal logistic regression) and flexible non-parametric machine learning-based analyses for all physical activity outcome variables. Importantly, these three approaches answer distinct research questions and evaluate different aspects of the data, rather than serving as equivalent evidence for a single conclusion. Bivariate analyses serve to describe crude associations between correlates and physical activity. The parametric linear and ordinal logistic regression models enable quantification and interpretation of adjusted associations. The flexible non-parametric machine learning models evaluate global predictive performance and identify feature importance, capturing complex non-linear interactions without assuming a rigid functional form. Overall, the combination of these approaches leverages unique methodological strengths to analyse the data from descriptive, inferential and predictive perspectives. Correlates selected by the regression models are evaluated alongside the predictive feature importances from the machine learning models to provide complementary insights into the underlying patterns of physical activity behaviour.

#### Bivariate analysis.

Bivariate analyses were conducted for all correlates of physical activity behaviour by calculating the appropriate association statistic (Pearson’s or Spearman’s correlation coefficient or Cohen’s d) with 95% confidence interval. Pearson’s correlation coefficient was used for approximately linear associations between continuous variables with approximately symmetric distributions, whereas Spearman’s rank correlation coefficient was used for ordinal variables. Cohen’s d was used to quantify differences between binary categorical variables and the physical activity outcomes.

#### Parametric regression analysis.

To study the relationship between correlates (independent variables, $X_{i},\;i=\text{1},\ldots k$) and the continuous physical activity outcomes (dependent, or target variable, Y) we used a multiple linear regression model. The analyses were conducted using the stats R package v4.2.3 in R statistical software v4.2.3 [35]. The linear model is defined by $Y=\beta_{\text{0}}+\beta_{\text{1}}X_{\text{1}}+\ldots +\beta_{k}X_{k}+\varepsilon$, with Y a continuous outcome variable and $\varepsilon \sim N(\text{0},\sigma^{\text{2}})$ a normally distributed error. $\beta_{i},\;i=\text{1},\ldots ,k$ are the regression coefficients and represent the expected change in outcome if $X_{i}$ changes by one unit and all other variables are held constant. However, the value of $\beta_{i}$ changes if the set of independent variables in the model changes and $X_{i}$ is correlated with other independent variables. Thus, the variable selection is of high relevance. To iteratively examine the statistical significance of each independent variable in the regression model, we considered a stepwise regression with bidirectional variable selection based on Akaike information criterion [36].

To study the ordinal physical activity outcomes, we used an ordinal logistic regression model (proportional odds model). The model was estimated using the polr() function from the MASS package in R. This approach assumes that the relationship between each predictor and the cumulative odds of being in a higher category of physical activity is constant across thresholds. Coefficients represent the log-odds of being in a higher activity category associated with one-unit increases (or changes) in the covariates, holding all other variables constant. Variance inflation factors were examined for the full regression models (before stepwise selection) and were consistently below 2, suggesting that multicollinearity was not a concern.

#### Non-parametric machine learning analysis.

To prepare the data for flexible prediction-oriented machine learning models, the categorical variables were converted into numerical values using a one-hot encoding technique via Python Pandas library. One-hot encoding represents categorical variables as binary vectors, where each category is converted into a new column, and a binary value (0 or 1) indicates the presence or absence of that category in the original data. Thereafter, the dataset was split into a train and test set (80/20 ratio using the scikit-learn library) [37] for building the machine learning models. In the modelling phase, we employed a set of traditional machine learning regression and classification (for CAI and RAI) models from the scikit-learn library [37], including decision trees (DecisionTreeRegressor/Classifier), support vector machines (SVM), k-nearest neighbours (KNeighborsRegressor/Classifier), random forest (RandomForestRegressor/Classifier) and gradient boosting (XGBRegressor/Classifier). The decision to use traditional machine learning models for exploration, as opposed to advanced deep learning approaches, was made given the relatively small size of the dataset. For the regression tasks, model performance was compared in terms of mean absolute error (MAE) and root mean squared error (RMSE) on the respective physical activity outcome variable. For the classification tasks for CAI and RAI, 5-fold cross-validation micro-averaged area under the receiver operating characteristic curve (AUC) scores using the One-vs-Rest (OvR) strategy were compared. The GridSearchCV method from scikit-learn [37] was used to find the optimal hyper-parameter for the aforementioned models. All models were finally evaluated on the test data set. We also used the Shapley Additive exPlanations (SHAP) framework [38] for understanding the importance of features (independent variables) within the best-performing model. The SHAP value offers a framework for interpreting the output of machine learning models and understanding the impact of each feature on the model's performance. Importantly, SHAP values should be interpreted as contributions to model prediction, not as evidence of causal or clinical importance.

## Results

**Fig 1:**
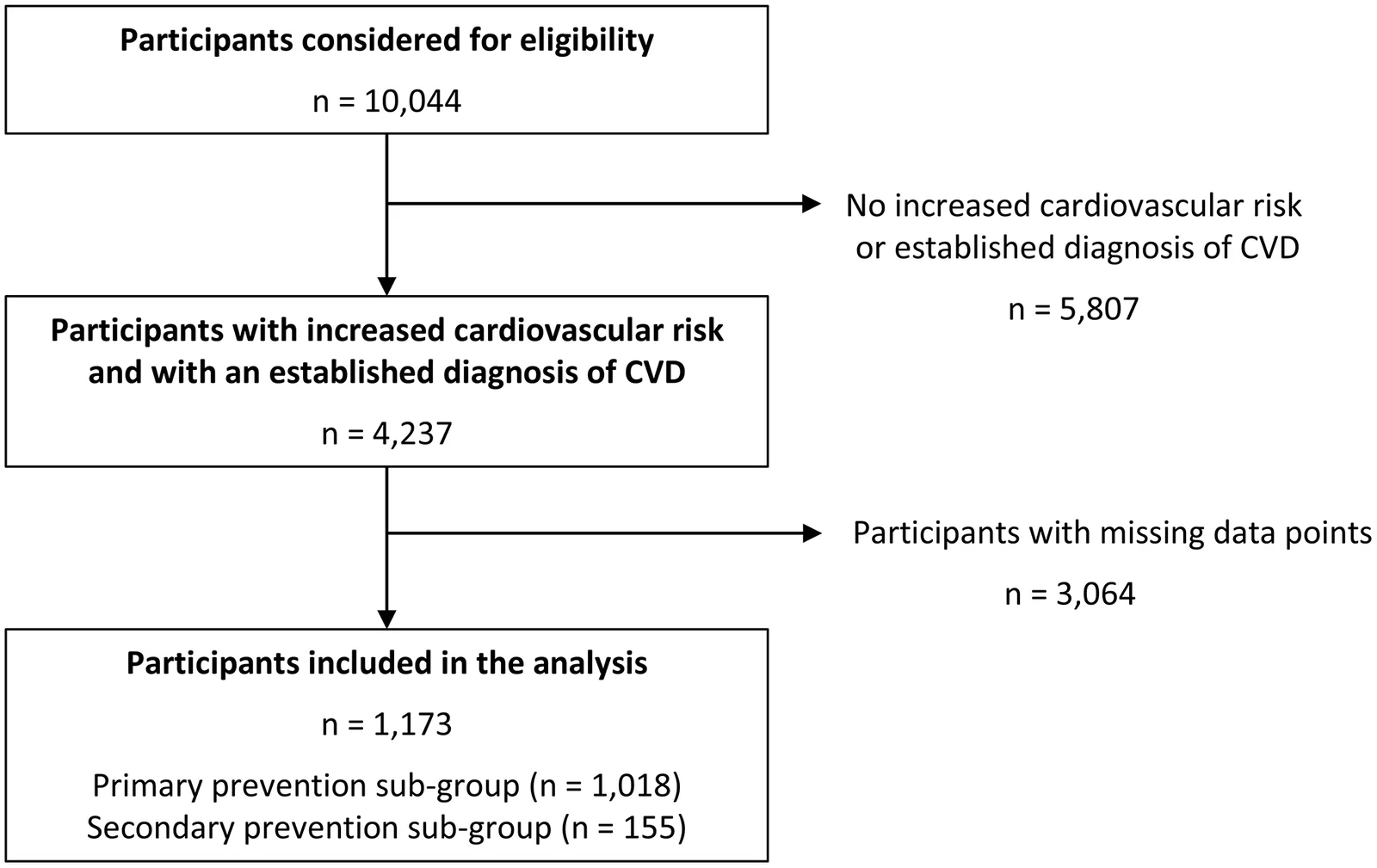
Participant flow.

Fig 1 describes the flow of participants through the study. Participants included in the analysis (n = 1,173) had a mean age of 60.5 years (SD 6.3, range 42–72), and 19% were female. Participants in the secondary prevention sub-group (n = 155) had diagnoses of coronary heart disease (39%), stroke (31%), atrial fibrillation (28%), chronic heart failure (7%), peripheral arterial disease (5%) and abdominal aortic aneurysm (3%). In the primary prevention group (n = 1,018), 82% of participants had high CVD risk (orange SCORE2/SCORE2-OP risk category) and 18% had very high CVD risk (red SCORE2/SCORE2-OP risk category). The primary physical activity outcome for the entire sample amounted to a mean of 13.9 (SD 11.6) weekly hours of walking, cycling and doing sports. Participant characteristics are summarised in Table 1.

**Table 1:** Participant characteristics of the entire sample and according to CVD risk group.

Characteristics		All n = 1173	High-risk primary prevention group n = 831	Very high-risk primary prevention group n = 187	Secondary prevention group n = 155
Age, (years) mean (SD)		60.5 (6.3)	59.4 (6.12)	64.7 (4.62)	61.4 (6.36)
Sex	Male	952 (81%)	659 (79%)	176 (94%)	117 (76%)
	Female	221 (19%)	172 (21%)	11 (6%)	38 (24%)
Education level	Low (ISCED 0–2)	65 (6%)	47 (6%)	13 (7%)	5 (3%)
	Medium (ISCED 3–5)	694 (59%)	477 (57%)	118 (63%)	99 (64%)
	High (ISCED 6–8)	402 (34%)	300 (36%)	55 (29%)	47 (30%)
	Other	12 (1%)	7 (1%)	1 (1%)	4 (3%)
Occupation	Blue-collar	122 (10%)	87 (10%)	25 (13%)	10 (7%)
	White-collar	858 (73%)	607 (73%)	127 (68%)	124 (80%)
	Self-employed	193 (17%)	137 (17%)	35 (19%)	21 (13%)
Retired	Yes	490 (42%)	284 (34%)	126 (67%)	80 (52%)
	No	683 (58%)	547 (66%)	61 (33%)	75 (48%)
Migration background	First generation	86 (7%)	59 (7%)	12 (6%)	15 (9%)
	Second generation	24 (2%)	17 (2%)	6 (3%)	1 (1%)
	Not first or second generation	1063 (91%)	755 (91%)	169 (91%)	139 (90%)
Religion	Yes	896 (76%)	632 (76%)	138 (74%)	126 (81%)
	No	277 (24%)	199 (24%)	49 (26%)	29 (19%)
Monthly household income after tax (Euros)	≤2000	297 (25%)	198 (24%)	47 (25%)	52 (34%)
	2001–4000	582 (50%)	407 (49%)	102 (54%)	73 (47%)
	>4000	218 (19%)	177 (21%)	22 (12%)	19 (12%)
	Not specified	76 (6%)	49 (6%)	16 (9%)	11 (7%)
Marital status	Living with partner	1060 (90%)	746 (90%)	175 (94%)	139 (90%)
	Separated or no partner	113 (10%)	85 (10%)	12 (6%)	16 (10%)
Number of children, median (range)		3 (1, 12)	3 (1, 9)	3 (1, 12)	3 (1, 5)
Comorbidity	0	647 (55%)	544 (65%)	103 (55%)	0
	1-2	452 (39%)	264 (32%)	73 (39%)	115 (74%)
	≥3	74 (6%)	23 (3%)	11 (6%)	40 (26%)
Years since first CVD diagnosis, median (range)		N/A	N/A	N/A	6.5 (<1, 52)
BMI, mean (SD)		27.5 (4.3)	27.3 (4.25)	28.4 (4.07)	27.6 (4.73)
Obesity (BMI > 30 kg/m2)	No	904 (77%)	654 (79%)	134 (72%)	116 (75%)
	Yes	269 (23%)	177 (21%)	53 (28%)	39 (25%)
WOMAC gonarthrosis score, mean (SD)		7.5 (21.5)	7.03 (20.6)	10.4 (27.5)	6.2 (17.4)
WOMAC coxarthrosis score, mean (SD)		3.8 (15.9)	3.5 (15.0)	4.9 (18.9)	4.3 (16.5)
ODI score, mean (SD)		5.6 (7.8)	5.4 (7.7)	5.6 (7.9)	6.5 (8.7)
BDI score, mean (SD)		3.8 (4.7)	3.6 (4.7)	3.4 (4.7)	4.8 (4.6)
ESS score, mean (SD)		6.9 (3.4)	7.0 (3.5)	6.6 (3.1)	6.6 (3.7)
BIS-15 score, mean (SD)		23.9 (4.0)	23.8 (4.1)	24.4 (3.8)	24.1 (3.9)
Smoking status	Never	475 (41%)	352 (42%)	63 (34%)	60 (39%)
	Former	466 (40%)	332 (40%)	60 (32%)	74 (48%)
	Current	232 (19%)	147 (18%)	64 (34%)	21 (13%)
AUDIT score, mean (SD)		4.3 (3.3)	4.4 (3.2)	4.5 (3.4)	3.9 (3.1)
Past physical activity behaviour, (average hours per week of doing sports) mean (SD)		3.0 (0.8)	3.0 (0.8)	3.0 (0.9)	2.9 (0.8)
Inventory on Mediterranean diet score, mean (SD)		6.3 (2.1)	6.3 (2.1)	6.3 (1.9)	6.7 (2.2)
Number of people in the household, median (range)		1 (1, 8)	1 (1, 6)	1 (1, 4)	1 (1, 8)
Type of housing	Multi-party house with 10 or more units	223 (19%)	143 (17%)	42 (23%)	38 (25%)
	Multi-party house with a maximum of 9 units	285 (24%)	203 (24%)	49 (26%)	33 (21%)
	Two-family terraced house	256 (22%)	191 (23%)	35 (19%)	30 (20%)
	Detached house	405 (34%)	290 (35%)	61 (32%)	54 (34%)
	other	4 (1%)	4 (1%)	0 (0%)	0
Size of living quarters, (m^2^) mean (SD)		114.4 (46.2)	115.0 (45.2)	114.0 (52.6)	112.0 (43.7)
Population density	Dense	609 (52%)	408 (49%)	115 (62%)	86 (55%)
	Intermediate	499 (42%)	386 (47%)	61 (33%)	52 (34%)
	Rural	65 (6%)	37 (4%)	11 (5%)	17 (11%)
Environmental noise, mean (SD)		1.3 (0.5)	1.3 (0.5)	1.3 (0.5)	1.4 (0.6)
Air pollution, mean (SD)		1.3 (0.5)	1.3 (0.5)	1.3 (0.5)	1.4 (0.6)
Physical activity^*^, mean (SD)	Walking, cycling & doing sports	13.9 (11.6)	13.9 (11.5)	14.0 (12.1)	13.9 (11.2)
	Gardening, doing handiwork/ repairs & housework	10.8 (11.1)	10.8 (11.2)	11.1 (11.6)	10.5 (9.6)
Cambridge Activity Index	Active	446 (38%)	327 (39%)	62 (33%)	57 (37%)
	Moderately active	308 (26%)	237 (29%)	58 (31%)	46 (30%)
	Moderately inactive	341 (29%)	222 (27%)	48 (26%)	38 (24%)
	Inactive	78 (7%)	45 (5%)	19 (10%)	14 (9%)
Recreational Activity Index	Active	516 (44%)	366 (44%)	83 (44%)	67 (43%)
	Moderately active	268 (23%)	193 (23%)	39 (21%)	36 (23%)
	Moderately inactive	183 (15%)	133 (16%)	26 (14%)	24 (16%)
	Inactive	206 (18%)	139 (17%)	39 (21%)	28 (18%)

AUDIT, Alcohol Use Disorder Identification Test; BDI, Beck Depression Inventory; BIS-15, Barratt Impulsiveness Scale; BMI, Body Mass Index; CVD, cardiovascular disease; ESS, Epworth Sleepiness Scale; ISCED, International Standard Classification of Education; N/A, not applicable; ODI, Oswestry Disability Index; WOMAC, Western Ontario and McMaster Universities Osteoarthritis Index

*Hours per week

### Primary physical activity outcome

The bivariate analysis for the primary physical activity outcome is presented in Table 2. In multiple regression analysis (Table 3), more past physical activity, being in retirement, adhering to the Mediterranean diet and increased air pollution showed statistically significant positive associations with physical activity behaviour. Statistically significant factors associated negatively with physical activity behaviour were a higher number of people living in the household, higher monthly household income, low mood/depression and lower occupancy housing. The factors included in the regression model explain approximately 15% of the variation in the outcome (adjusted R-squared 0.145). The residuals plot showed random distribution of residuals with no discernible pattern, providing no indication against adequate model fit.

**Table 2:** Bivariate association statistics for the primary physical activity outcome (weekly hours of walking, cycling and doing sports).

Category	Variable	N; mean (SD) / median (range)	Physical activity, mean (SD)	Association with Physical activity (95% CI)	P-value	Association statistic
Socio-demographic	Age	1173 (100%); 60.5 (6.3)	–	0.11 (0.05, 0.16)	<0.01	r
	Sex	Male: 952 (81%)	13.9 (11.4)	0.03 (−0.11, 0.18)	0.66	d
		Female: 211 (19%)	14.2 (12.4)			
	Education level	Low (ISCED 0–2): 65 (6%)	15.4 (12.0)	−0.08 (−0.33, 0.17)	0.54	d
		Medium (ISCED 3–5): 694 (59%)	14.3 (12.6)			
		Low (ISCED 0–2): 65 (6%)	15.4 (12.0)	−0.25 (−0.51, 0.01)	0.06	d
		High (ISCED 6–8): 402 (34%)	12.9 (9.32)			
		Medium (ISCED 3–5): 694 (59%)	14.3 (12.6)	−0.13 (−0.25, 0.00)	0.05	d
		High (ISCED 6–8): 402 (34%)	12.9 (9.32)			
	Occupation	Blue-collar: 122 (10%)	11.7 (12.5)	−0.01 (−0.24, 0.21)	0.91	d
		Self-employed: 193 (17%)	11.6 (8.12)			
		Blue-collar: 122 (10%)	11.7 (12.5)	0.25 (0.06, 0.44)	0.01	d
		White-collar: 858 (73%)	14.8 (12.0)			
		Self-employed: 193 (17%)	11.6 (8.12)	0.28 (0.12, 0.43)	<0.01	d
		White-collar: 858 (73%)	14.8 (12.0)			
	Migration background	First generation: 86 (7%)	10.9 (8.82)	0.37 (−0.08, 0.83)	0.11	d
		Second generation: 24 (2%)	14.3 (11.2)			
		First generation: 86 (7%)	10.9 (8.82)	0.29 (0.07, 0.51)	0.01	d
		Not first or second generation: 1063 (91%)	14.2 (11.7)			
		Second generation: 24 (7%)	14.3 (11.2)	−0.01 (−0.42, 0.39)	0.95	d
		Not first or second generation: 1063 (91%)	14.2 (11.7)			
	Religion	Yes: 896 (76%)	14.0 (12.2)	0.01 (−0.13, 0.14)	0.9	d
		No: 277 (24%)	13.9 (11.4)			
	Monthly household income after tax (Euro)	≤2000: 297 (25%)	14.9 (13.6)	−0.06 (−0.02, 0.08)	0.36	d
		2001 to 4000: 582 (50%)	14.1 (11.2)			
		≤2000: 297 (25%)	14.9 (13.6)	−0.23 (−0.41, −0.06)	0.01	d
		>4000: 218 (19%)	12.1 (9.04)			
		2001 to 4000: 582 (50%)	14.1 (11.2)	−0.18 (−0.34, −0.03)	0.02	d
		>4000: 218 (19%)	12.1 (9.04)			
	Marital status	Living with partner: 1060 (90%)	14.0 (11.4)	−0.03 (−0.22, 0.17)	0.78	d
		Separated or no partner: 113 (10%)	13.6 (13.3)			
	Number of children	1173 (100%); 3 (1, 12)	–	−0.04 (−0.10, 0.01)	0.17	rs
	Retirement	No: 683 (58%)	12.67(11.6)	0.26 (0.14, 0.37)	<0.01	d
		Yes: 490 (42%)	15.6 (11.3)			
Clinical	CVD risk group	Primary high: 831 (71%)	13.9 (11.5)	0.01 (−0.15, 0.17)	0.93	d
		Primary very high: 187 (16%)	14.0 (12.1)			
		Primary high: 831 (71%)	13.9 (11.5)	0 (−0.17, 0.17)	0.99	d
		Secondary: 155 (13%)	13.9(11.2)			
		Primary very high: 187 (16%)	14.0 (12.1)	−0.01 (−0.22, 0.21)	0.95	d
		Secondary: 155 (13%)	13.9 (11.2)			
	Comorbidity	0: 647 (55%)	14.0 (11.1)	−0.02 (−0.14, 0.1)	0.71	d
		1-2: 452 (39%)	13.7 (11.8)			
		0: 647 (55%)	14.0 (11.1)	0.03 (−0.21, 0.27)	0.82	d
		3 + : 74 (6%)	14.3 (13.4)			
		1-2: 452 (39%)	13.7 (11.8)	0.05 (−0.2, 0.29)	0.7	d
		3 + : 74 (6%)	14.3 (13.4)			
	Body mass index	1173 (100%); 27.5 (4.3)	–	−0.09 (−0.14, −0.03)	<0.01	r
	Knee pain	1173 (100%); 0 (0, 144)	–	0.08 (0.03, 0.14)	<0.01	rs
	Hip pain	1173 (100%); 0 (0, 145)	–	0.06 (−0.00, 0.11)	0.05	rs
	Low back pain	1173 (100%); 2 (0, 50)	–	−0.05 (−0.11, 0.01)	0.08	rs
Psychological/ cognitive	Depression	1173 (100%): 2 (0, 39)	–	−0.10 (−0.16, −0.04)	<0.01	rs
	Daytime sleepiness	1173 (100%); 7 (0, 20)	–	−0.02 (−0.08, 0.03)	0.43	rs
	Impulsivity	1173 (100%); 24 (15, 39)	–	−0.04 (−0.10, 0.01)	0.16	rs
Lifestyle	Smoking status	Former: 466 (40%)	14.8 (11.4)	−0.08 (−0.21, 0.05)	0.23	d
		Never: 475 (41%)	13.9 (10.8)			
		Former: 466 (40%)	14.8 (11.4)	−0.4 (−0.2, −0.04)	0.01	d
		Current: 232 (19%)	12.4 (13.1)			
		Never: 475 (41%)	13.9 (10.8)	−0.3 (−0.13, 0.03)	0.1	d
		Current: 232 (19%)	12.4 (13.1)			
	Alcohol use	1173 (100%); 4 (0, 26)	–	−0.02 (−0.08, 0.04)	0.52	rs
	Past physical activity behaviour (hours per week of doing sports at ages 5–19, 20–34, 35–49, and 50+)	1173 (100%); 3.0 (0.8)	–	0.39 (0.34, 0.43)	<0.01	rs
	Mediterranean diet	1173 (100%); 6.3 (2.1)	–	0.15 (0.10, 0.21)	<0.01	rs
Environmental	Number of people in household	1173 (100%); 1 (1, 8)	–	−0.10 (−0.16, −0.04)	<0.01	rs
	Housing	Detached house: 405 (34%)	14.1 (11.0)	−0.23 (−0.39, −0.08)	<0.01	d
		Two-family terraced house: 256 (22%)	11.7 (9.2)			
		Detached house: 405 (34%)	14.1 (11.0)	0.08 (−0.07, 0.24)	0.28	d
		Multi-party house with a maximum of 9 units: 285 (24%)	15.1 (13.6)			
		Detached house: 405 (34%)	14.1 (11.0)	0.07 (−0.09, 0.23)	0.41	d
		Multi-party house with 10 or more units: 223 (19%)	14.9 (11.9)			
		Two-family terraced house: 256 (22%)	11.7 (9.2)	0.29 (0.12, 0.46)	<0.01	d
		Multi-party house with a maximum of 9 units: 285 (24%	15.1 (13.6)			
		Two-family-terraced house: 256 (22%)	11.7 (9.2)	0.3 (0.12, 0.48)	<0.01	d
		Multi-party house with 10 or more units: 223 (19%)	14.9 (11.9)			
		Multi-party house with a maximum of 9 units: 285 (24%)	15.1 (13.6)	−0.02 (−0.19, 0.16)	0.84	d
		Multi-party house with 10 or more units: 223 (19%)	14.9 (11.9)			
	Size of living quarters (m^2^)	1173 (100%); 114.4 (46.2)	–	−0.06 (−0.12, −0.0)	0.05	r
	Population density	Dense: 609 (52%)	13.7 (10.9)	0.01 (−0.11, 0.13)	0.83	d
		Intermediate: 499 (42%)	13.9 (11.7)			
		Dense: 609 (52%)	13.7 (10.9)	0.25 (0.00, 0.51)	0.05	d
		Rural: 65 (6%)	16.6 (15.6)			
		Intermediate: 449 (42%)	13.6 (11.4)	0.22 (−0.04, 0.48)	0.09	d
		Rural: 65 (6%)	15.9 (15.2)			
	Environmental noise	1173 (100%); 1.3 (0.5)	–	0.02 (−0.04, 0.08)	0.52	rs
	Air pollution	1173 (100%); 1.3 (0.5)	–	0.07 (0.01, 0.13)	0.02	rs

CVD, cardiovascular disease

d, Cohen’s d

r, Pearson’s correlation coefficient

rs, Spearman’s rank correlation coefficient

**Table 3:** Multiple linear stepwise regression for the primary physical activity outcome (weekly hours of walking, cycling and doing sports).

Coefficients	Estimate	SE	t-value	Pr(>|t|)
(Intercept)	3.09126	3.07239	1.006	0.3146
Past physical activity behaviour (doing sports)	4.09663	3.07239	10.325	**<0.0001**
Retirement	1.49725	0.69323	2.160	**0.0310**
Mediterranean diet	0.47236	0.15723	3.004	**0.0027**
Occupation: self-employed	−1.20541	1.28274	−0.940	0.3476
Occupation: white-collar	1.71205	1.09376	1.565	0.1178
Number of people in household	−0.81742	0.37188	−2.198	**0.0281**
Monthly household income: 2001–4000	−1.18165	0.79954	−1.478	0.1397
Monthly household income: > 4000	−3.00283	1.03962	−2.888	**0.0039**
Monthly household income: not specified	−0.94484	1.38746	−0.681	0.4960
Depression	−0.16118	0.06806	−2.368	**0.0180**
Air pollution	2.05622	0.73394	2.802	**0.0052**
Housing: Two-family-terraced house	−2.27793	0.85838	−2.654	**0.0081**
Housing: Multi-party house with a maximum of 9 units	0.27805	0.83908	0.331	0.7404
Housing: Multi-party house with 10 or more units	0.30655	0.91526	0.335	0.7377
Housing: Others	−1.42316	0.91526	−0.262	0.7932
Body Mass Index	−0.13403	0.07500	−1.787	0.0742
Environmental noise	−1.27320	0.07500	−1.739	0.0824

Residual standard error: 10.69 on 1155 degrees of freedom

Multiple R-squared: 0.1575. Adjusted R-squared: 0.1451

F-statistic: 12.7 on 17 and 1155 DF, p-value < 0.001

**Fig 2:**
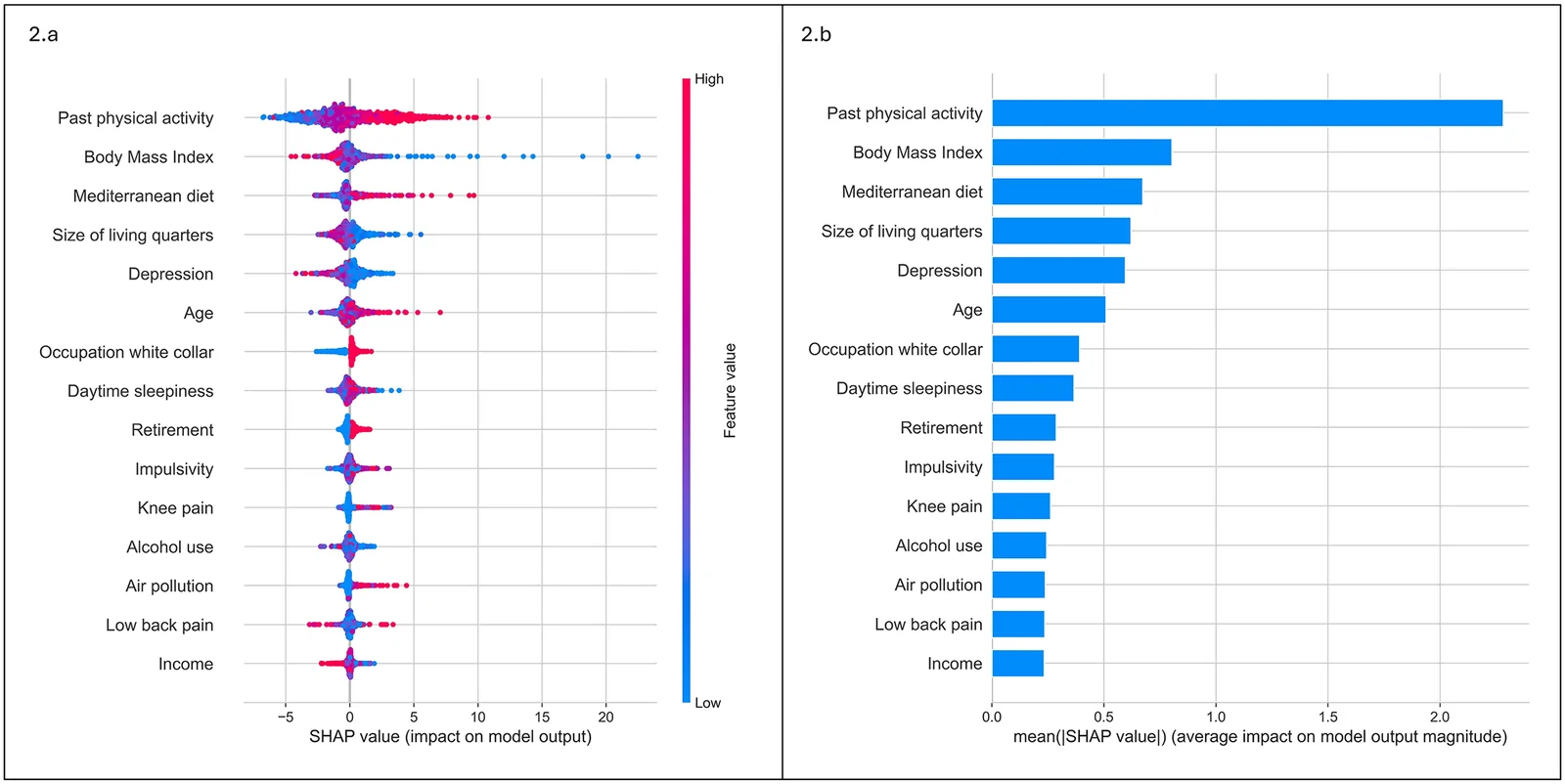
Shapley Additive exPlanations (SHAP) plot of the XGBRegressor model. Shown are the top 15 features contributing to the model prediction and their relative contribution. Fig 2a shows the direction of the features’ (independent variables’) impact on the model output, e.g., higher past physical activity (red data points) predominantly contributing to higher physical activity (positive portion of the x-axis). Fig 2b shows the average impact of individual features on the model output magnitude relative to each other.

Performance indicators (MAE and RMSE) for the five machine learning models are shown in Table 4. These indicate similar performance of all five models, with XGBRegressor showing slightly superior performance (lowest MAE and RMSE comparable to the RandomForestRegressor). We therefore present the feature importance analysis for the XGBRegressor model (Fig 2). The Shapley Additive exPlanations (SHAP) plot (Fig 2a) shows how the top 15 features (correlates) contribute to the model performance. The feature value is indicated by the colour spectrum blue (lower value) to red (higher value). The direction the feature impacts on the outcome is indicated on the x-axis (positive or negative association). The features are ranked in order of importance from top to bottom (average impact of individual features on the model output magnitude, Fig 2b).

**Table 4:** Performance of five machine learning models in prediction of the primary physical activity outcome (weekly hours of walking, cycling and doing sports).

Model	MAE	RMSE
XGBRegressor	7.31	10.11
RandomForestRegressor	7.40	10.15
SVM	7.69	11.00
DecisionTreeRegressor	8.24	10.87
KNeighborsRegressor	7.93	10.57

MAE, mean absolute error

RMSE, root mean squared error

SVM, support vector machine

In machine learning analysis, past physical activity behaviour, Mediterranean diet, age, white collar occupation and retirement are the five positively associated features that contribute most to the model. Among the top 15 features of the XGBRegressor model, past physical activity, Body Mass Index, Mediterranean diet, depression, occupation, retirement, air pollution and income are also represented in the regression model, whereas size of living quarters, age, daytime sleepiness, impulsivity, knee pain, alcohol use and low back pain are included in the machine learning model, but not in the regression model.

### Secondary physical activity outcomes

Detailed results of the regression and machine learning analyses for the secondary physical activity outcomes are given in supporting information S1 File, whereby the machine learning analyses largely align with the results from the multiple regression models. Comparing the primary physical activity outcome against CAI and RAI, the factors past physical activity behaviour, Mediterranean diet and low mood/depression are retained in the multiple regression models for both CAI and RAI. Higher income is corroborated in the model for CAI, whereas lower occupancy housing is corroborated in the model for RAI. Retirement is corroborated in the model for RAI but not retained in the model for CAI. Additional statistically significant factors in these models are current smoking (associated with less physical activity), greater comorbidity (associated with less physical activity for RAI), higher body mass index (associated with less physical activity for CAI) and self-employed occupation (associated with less physical activity for CAI).

In the comparison of the primary physical activity outcome (representing more leisure-type physical activities, or activities outside the home) against gardening, doing handiwork/repairs and housework (representing chores and maintenance-type physical activities in and around the home) and against total physical activity, several factors are retained (retirement, Mediterranean diet, low mood/depression, income, housing), but two differences are noteworthy: past physical activity is not retained in the multiple regression model for gardening, doing handiwork/repairs and housework, but remains in the model for total physical activity; and female sex is included in both models as a factor that is associated with more physical activity.

## Discussion

This is the first study to analyse correlates of physical activity behaviour in a sample with increased cardiovascular risk and established CVD in Austria. Although there are some differences between the correlates included in the multiple regression analyses and in the machine learning models, both analytic approaches describe an overall coherent picture of these factors’ associations with physical activity behaviour. The main message derived from the combined analyses is that, in this age group – spanning the second half of working age to early retirement, i.e., 40–70 years – those who were retired, those who had been more physically active during earlier life decades, and those who adhered more to a Mediterranean diet were more physically active. Additional correlates were retained in some but not all the models, such as the inverse association of comorbidity and depression with physical activity, which nevertheless describe coherent trends. The inverse association between comorbidity and physical activity, for example, is well-documented in the literature for general population samples (e.g., [39]) and CVD groups (e.g., [40]) alike. Sex was not associated with the primary physical activity outcome (walking, cycling and doing sports), but female sex was associated with more physical activity for both total physical activity and gardening, doing handiwork/repairs and housework, whereby the association in total physical activity was driven by the difference between sexes in gardening, doing handiwork/repairs and housework.

When interpreting these findings, it is important to bear in mind the limitations inherent to a cross-sectional dataset. These findings represent correlations, not determinants, of physical activity behaviour and serve an exploratory or hypothesis-generating purpose. Longitudinal or interventional evidence is required to demonstrate causal relationships. Moreover, in a cross-sectional dataset the possibility of reverse causation needs to be considered, particularly for clinical variables such as body mass index, pain, comorbidity and depression, which may both influence and be influenced by physical activity.

### Retirement

Our main findings largely align with other literature on correlates of physical activity behaviour. Several studies have documented that the life transition to retirement often leads to a more physically active lifestyle in the general population. A recent systematic review [41] included 36 longitudinal studies from Australia, Europe, Japan and the United States, showing a consistent increase in leisure time physical activity following retirement. The authors suggest that the absence of work activities provides retirees with an opportunity for leisure activities, including physical activity. Pooled longitudinal data [42] from 106,927 individuals across 35 countries in East Asia, Europe, North and Central America revealed that physical inactivity in retirement decreased on average by 3 percentage points (−0.03, 95% CI −0.05 to −0.01). However, studies also indicate a simultaneous increase in sedentary time, and a reduction in work- and transport-related physical activity [41]. Moreover, change in physical activity following retirement seems to be moderated by socio-demographic factors, whereby lower socio-economic positions and retirement from physically demanding occupations are associated with decrease in physical activity [41]. Contrary to this, our analysis gives some indication of an inverse relationship between socio-demographic markers (income, housing) and physical activity. Specific to CVD populations, analyses of data from 32,370 outpatients with stable coronary artery disease [40] resulted in an inverse association, with those in retirement versus full-time employment less likely to engage in vigorous physical activity (odds ratio 0.70, 95% CI 0.65 to 0.74). But this finding may not be directly comparable to our data, as our physical activity outcomes captured physical activity across all intensity levels.

The model for CAI partly contradicts the pictures presented in the other models, because it excludes retirement. This is explained by an assumption included in the formula for CAI [33]. CAI is derived from weekly hours of cycling and doing sports, plus factoring in occupational physical activity (sedentary, light, moderate or heavy manual labour). For retirees, the question about occupational physical activity does not apply and is therefore assumed to be sedentary in the formula for CAI. This assumption accounts for the elimination of the impact of retirement in the model for CAI and should be discarded.

### Past physical activity behaviour

Our study corroborates positive associations between past and current physical activity behaviour that have also been observed by others. A cohort study of 712 healthy World War II veterans in the United States, for example, found a statistically significant correlation (Spearman’s rho 0.164, p < 0.01) between participation in high school sports and physical activity at age 70 + years [43]. Similarly, a general population cohort of 2,201 Australians showed some statistically significant weak correlations between childhood and adult physical activity [44]. In a cohort of 105 Swedes, physical activity during adolescence in addition to other physical performance characteristics explained 82% of the physical activity level in adulthood for women and 47% for men [45].

In CVD samples, several studies have demonstrated short-term associations between prior and current physical activity. In a study of 114 patients with myocardial infarction, physical activity approximately one week after the event correlated with physical activity 8 months after the event (Pearson’s r 0.34, 95% CI 0.17 to 0.49) [46]. In 200 patients with coronary artery disease, walking behaviour two weeks after percutaneous coronary intervention correlated with walking behaviour 6 months after the procedure (Pearson’s r 0.30, 95% CI 0.17 to 0.42) [47]. And a study of 801 patients with coronary heart disease reported a correlation in physical activity from 6 months to 12 months after hospitalisation (Pearson’s r 0.61, 95% CI 0.56 to 0.66) [48]. In contrast to these short-term correlations, previous physical activity in our study captured physical activity (specifically doing sports) across the preceding life decades, adding a long-term perspective to this literature in CVD populations.

### Mediterranean diet

The emergence of Mediterranean diet as a consistently associated factor with physical activity in our analysis adds a novel finding to the literature. While the Mediterranean diet is recognised as one of the best dietary strategies for the prevention of chronic diseases and premature death [49], and there is evidence to support the synergistic effects of a Mediterranean diet combined with recommended levels of physical activity [49], we are not aware of reports of this association in a sample with increased cardiovascular risk or established CVD. In the general population, studies have found that more physical activity and healthier eating habits correlate to a certain extent, but individual heterogeneity can be considerable (e.g., [50]). In the context of our study, we suggest that both previous physical activity and Mediterranean diet could be interpreted as indicators of a person’s greater health awareness and health literacy, resulting in a more health-conscious lifestyle with higher levels of physical activity.

### Gender

Contrary to our findings, female sex is most frequently reported to be negatively associated with physical activity, for example in an umbrella review [51] synthesising 11 reviews representing over 300 unique primary papers on physical activity in community-dwelling older adults. In CVD populations, female sex was an independent predictor of lower physical activity levels [40]. In a scoping review on physical activity post cardiac surgery [52] men were noted to be more physically active than women, generally walking at a faster pace and exerting more effort during exercise. Our contrary finding could be idiosyncratic to the geographic region the sample was recruited from. Austrian population statistics show that in the County of Salzburg the World Health Organisation’s physical activity recommendations are met by 26.8% and 13.9% of men in the age groups 45–59 and 60–74 years, respectively, compared to 28.5% and 25.4% of women in these respective age groups [53]. The strong association of female sex with more physical activity related to gardening, doing handiwork/repairs and housework might reflect traditional gender roles and cultural norms in this age group and geographic region of Austria.

In summary, our analysis indicates that the transition to retirement might offer an opportunity to enact a more physically active and heart-healthy lifestyle, which could be leveraged by targeted interventions for the primary and secondary prevention of CVD. Moreover, our findings might be interpreted to support the crucial importance of physical activity promotion, from early childhood and throughout the life course, as greater physical activity during earlier life decades likely facilitates the maintenance of physical activity during later life decades, also in individuals with CVD. With respect to the assessment of physical activity, our findings highlight the importance of capturing a broad range of physical activity behaviours, including gardening and household work, particularly for women, as our analysis shows that increased activities in these areas was associated with female sex. Lastly, the positive association between Mediterranean diet and physical activity presents a novel finding, and the relationship between dietary habits and physical activity could find further consideration in future research.

### Strengths and limitations

The P10 study is a unique dataset in the Austrian geographic region, due to the recruitment of a large population sample and the collection of a broad range of measurements and self-report questionnaires. This can be regarded as a strength of our analysis, allowing us to examine correlates which are less commonly captured in studies of physical activity in CVD samples such as impulsiveness, daytime sleepiness, past physical activity behaviour, Mediterranean diet, air pollution, and environmental noise. Another strength of our analysis was the investigation through both regression and machine learning analyses. While regression models allow us to quantify and interpret associations, the inclusion of machine learning models provide an additional perspective by capturing potential non-linear relationships. Importantly, the overlap in key correlates identified by both approaches strengthens the robustness of our findings and increases confidence in the observed associations.

Several limitations to our study are acknowledged. Self-reported measures of physical activity are inherently subject to recall and social desirability bias. However, the EPIC physical activity questionnaire was validated in large European cohorts [32,33], and the volumes of physical activity reported in P10 are in line with device-measured physical activity reported in other studies of adults in the same age range [54]. In the inclusion criteria, we prioritised the interpretation of the relative importance of correlates and the comparison of different physical activity outcomes. We therefore included only those participants with complete datasets, excluding 72% of P10 participants with increased cardiovascular risk or established diagnosis of CVD. Nevertheless, the sample size was considered adequate for the planned multiple regression analyses relative to the number of candidate variables included in the regression models and is consistent with commonly cited heuristic sample size rules for multiple regression [55].

Participant characteristics of the excluded group are provided in supporting information S2 File. In comparison with participants included in this analysis, the excluded group has a higher proportion of women (31% versus 19%) and higher proportions of participants with the lowest education level (11% versus 6%) and the lowest income category (39% versus 25%). These differences are acknowledged as a potential source of selection bias. Of note, underrepresentation of participants with lower socio-economic status (International Standard Classification of Education levels 0–5 and blue-collar employment) was also observed for the entire P10 study sample in comparison with population statistics for the city of Salzburg [7].

We acknowledge that this analysis was reliant on the variables available within the P10 dataset. The dataset lacked a number of correlates of physical activity that are well-documented in the literature, including individual cognitive/psychological factors such as self-efficacy and intention [6,56], interpersonal factors such as social support and loneliness [51,57] and environmental factors such as the built environment [58]. We suggest that this accounts for the large proportion of the outcome variation that was not explained by our analysis models. In addition, we acknowledge that the bidirectional stepwise regression approach used for variable selection has recognised limitations, including potential model instability and sensitivity to correlations among candidate variables. Our aim, however, was not to develop an optimal prediction model but to identify a parsimonious set of correlates associated with physical activity in this exploratory analysis. Future studies could evaluate alternative penalised regression approaches, such as LASSO, ridge regression or elastic net, to assess the robustness of variable selection.

## Conclusions

In this Austrian cohort of individuals with high cardiovascular risk or established diagnosis of CVD, retirement, more physical activity during earlier life decades and adherence to a Mediterranean diet were positively associated with physical activity behaviour, while greater comorbidity and depression were inversely associated with physical activity behaviour. These findings add to our understanding of correlates of physical activity behaviour in CVD populations and offer approaches for more selective prevention and intervention, e.g., leveraging the transition to retirement for physical activity promotion, or directing additional and tailored physical activity promotion offers towards those not adhering to a Mediterranean diet, those with a history of physical inactivity and those with greater comorbidity.

## Supporting information

S1 File Regression models and machine learning models for the secondary physical activity outcomes. (DOCX)

S2 File Characteristics of Paracelsus 10.000 participants with increased cardiovascular risk and established cardiovascular disease: sample included in the analysis (n = 1173) versus excluded participants (n = 3064). (DOCX)
