# Supplementary material for: Correlates of physical activity behaviour in a population sample with increased cardiovascular risk and established cardiovascular disease: Cross-sectional analysis of data from the Paracelsus 10,000 prospective cohort study in Salzburg, Austria
Source: PLoS One. 2026 Aug 19;21(8):e0356607. doi: 10.1371/journal.pone.0356607 (PMC13489455; doi:10.1371/journal.pone.0356607)
Supplement: Supplementary file 1 [file pone.0356607.s001.docx]

**S1 Supporting Information.** **Regression models and machine learning models for the secondary physical activity outcomes.**

Content

[1. Physical activity outcome variable: Cambridge Activity Index (CAI), N = 1,173	2](#_Toc224563657)

[1.1. Multiple ordinal logistic regression	2](#_Toc224563658)

[1.2. Machine learning model	3](#_Toc224563659)

[2. Physical activity outcome variable: Recreational Activity Index (RAI), N = 1,173	5](#_Toc224563660)

[2.1. Multiple ordinal logistic regression	5](#_Toc224563661)

[2.2. Machine learning model	6](#_Toc224563662)

[3. Physical activity outcome variable: Weekly hours of gardening, doing handiwork/repairs and housework, N = 1,173	8](#_Toc224563663)

[3.1. Multiple linear regression	8](#_Toc224563664)

[3.2. Machine learning model	9](#_Toc224563665)

[4. Physical activity outcome variable: Total physical activity (weekly hours of walking, cycling, doing sports, gardening, doing handiwork/repairs and housework), N = 1,173	11](#_Toc224563666)

[4.1. Multiple linear regression	11](#_Toc224563667)

[4.2. Machine learning model	12](#_Toc224563668)

# 1. Physical activity outcome variable: Cambridge Activity Index (CAI), N = 1,173

## 1.1. Multiple ordinal logistic regression

Coefficients	Estimate	Std. Error	t value	Pr(>|t|)

Age	-0.0173151	0.0096757	-1.7895	0.0737898 .

Female sex	-0.2316333	0.1622955	-1.4272	0.1537836

Body Mass Index	-0.0563501	0.0139848	-4.0294	5.959e-05 ***

Depression	-0.0452005	0.0125763	-3.5941	0.0003392 ***

Knee pain	0.0052320	0.0027416	1.9084	0.0565862 .

Smoking status: Never smoked	-0.0744824	0.1264858	-0.5889	0.5560707

Smoking status: Current smoker	-0.4982093	0.1667018	-2.9886	0.0028617 **

Occupation: Self-employed	-0.6832953	0.2304611	-2.9649	0.0030900 **

Occupation: White-collar	-0.3452567	0.1990727	-1.7343	0.0831276 .

Religion	0.1961036	0.1335658	1.4682	0.1423177

Income: 2001 to 4000 Euro	-0.3099898	0.1486684	-2.0851	0.0372788 *

Income: >4000 Euro	-0.6753505	0.1845332	-3.6598	0.0002638 ***

Income: Not specified	-0.1615190	0.2601269	-0.6209	0.5347723

Past physical activity behaviour	1.0850251	0.0778809	13.9318	< 2.2e-16 ***

Air pollution	0.1637176	0.1124347	1.4561	0.1456333

Mediterranean diet	0.0579337	0.0293494	1.9739	0.0486280 *

Signif. codes: 0 ‘***’ 0.001 ‘**’ 0.01 ‘*’ 0.05 ‘.’ 0.1 ‘ ’ 1

McFadden’s pseudo R²: 0.10

## 1.2. Machine learning model

Model performance was evaluated using 5-fold cross-validation. For each fold, we computed the micro-averaged ROC AUC using the One-vs-Rest (OvR) strategy for multi-class classification. The final score was reported as the mean and standard deviation across the five folds.

| Model | ROC AUC Score (Mean ± SD) |
| --- | --- |
| XGBClassifier | 0.73 ± 0.02 |
| RandomForestClassifier | 0.73 ± 0.01 |
| SVM | 0.73 ± 0.01 |
| KNeighborsClassifier | 0.69 ± 0.01 |
| DecisionTreeClassifier | 0.64 ± 0.02 |

SHAP analysis based feature importance (top 15 features) for CAI classification/prediction by XGBClassifier. The SHAP values are aggregate of all 5-folds of cross validation.

# 2. Physical activity outcome variable: Recreational Activity Index (RAI), N = 1,173

## 2.1. Multiple ordinal logistic regression

Coefficients	Estimate	Std. Error	t value	Pr(>|t|)

Comorbidities: 1-2	-0.146127	0.131595	-1.1104	0.2670442

Comorbidities: 3+	-0.658984	0.253485	-2.5997	0.0094499 **

Body Mass Index	-0.025581	0.015352	-1.6663	0.0959274 .

Depression	-0.033598	0.012650	-2.6559	0.0080178 **

Smoking status: Never smoked	-0.335401	0.129084	-2.5983	0.0094876 **

Smoking status: Current smoker	-0.541789	0.159986	-3.3865	0.0007318 ***

Occupation: Self-employed	-0.148950	0.223370	-0.6668	0.5050139

Occupation: White-collar	0.300649	0.190341	1.5795	0.1144891

Housing: Two-family-terraced house	-0.376465	0.151812	-2.4798	0.0132872 *

Housing: Multi-party house with a maximum of 9 units	-0.090131	0.150896	-0.5973	0.5504235

Housing: Multi-party house with 10 or more units	0.201526	0.168320	1.1973	0.2314448

Housing: Others	-0.365474	1.055740	-0.3462	0.7292721

Retirement	0.615611	0.124000	4.9646	7.922e-07 ***

Past physical activity behaviour	1.015661	0.076730	13.2368	< 2.2e-16 ***

Environmental noise	-0.222607	0.134276	-1.6578	0.0976235 .

Air pollution	0.253221	0.134991	1.8758	0.0609294 .

Mediterranean diet	0.071467	0.028830	2.4789	0.0133207 *

Signif. codes: 0 ‘***’ 0.001 ‘**’ 0.01 ‘*’ 0.05 ‘.’ 0.1 ‘ ’ 1

McFadden’s pseudo R²: 0.10

## 2.2. Machine learning model

| Model | ROC AUC Score (Mean ± SD) |
| --- | --- |
| XGBClassifier | 0.71 ± 0.02 |
| RandomForestClassifier | 0.71 ± 0.01 |
| SVM | 0.71 ± 0.01 |
| KNeighborsClassifier | 0.68 ± 0.01 |
| DecisionTreeClassifier | 0.63 ± 0.03 |

SHAP analysis based feature importance (top 15 features) for RAI classification/prediction by XGBClassifier. The SHAP values are aggregate of all 5-folds of cross validation.

# 3. Physical activity outcome variable: Weekly hours of gardening, doing handiwork/repairs and housework, N = 1,173

## 3.1. Multiple linear regression

Coefficients	Estimate	Std. Error	t value	Pr(>|t|)

(Intercept)	5.74516	2.45622	2.339	0.019503 *

Female sex	7.63246	0.83060	9.189	< 2e-16 ***

Retirement	2.87480	0.63035	4.561	5.64e-06 ***

Income: 2001 to 4000 Euro	-2.57961	0.75464	-3.418	0.000652 ***

Income: >4000 Euro	-4.21442	0.96376	-4.373	1.34e-05 ***

Income: Not specified	-4.09860	1.30059	-3.151	0.001667 **

Mediterranean diet	0.62836	0.14918	4.212	2.73e-05 ***

Low back pain	0.10179	0.04058	2.508	0.012265 *

Housing: Two-family-terraced house	-2.03356	0.80271	-2.533	0.011429 *

Housing: Multi-party house with a maximum of 9 units	-1.40490	0.77759	-1.807	0.071062 .

Housing: Multi-party house with 10 or more units	-2.35957	0.85090	-2.773	0.005643 **

Housing: Others	-3.31217	5.07545	-0.653	0.514153

Religion	-1.32850	0.69416	-1.914	0.055890 .

Depression	-0.11578	0.06690	-1.731	0.083774 .

Body Mass Index	0.10520	0.07072	1.488	0.137138

Signif. codes: 0 ‘***’ 0.001 ‘**’ 0.01 ‘*’ 0.05 ‘.’ 0.1 ‘ ’ 1

Residual standard error: 10.02 on 1158 degrees of freedom

Multiple R-squared: 0.1928,	Adjusted R-squared: 0.1831

F-statistic: 19.76 on 14 and 1158 DF, p-value: < 2.2e-16

## 3.2. Machine learning model

| **Model** | **MAE** | **RMSE** |
| --- | --- | --- |
| XGBRegressor | 6.55 | 9.56 |
| RandomForestRegressor | 6.65 | 9.58 |
| SVM | 7.04 | 11.04 |
| DesisionTreeRegressor | 7.56 | 10.49 |
| KNneighborsRegressor | 7.16 | 10.42 |
| MAE: mean absolute error  RMSE: root mean squared error  SVM: support vector machines | | |

Top 15 features by XGBRegressor model

# 4. Physical activity outcome variable: Total physical activity (weekly hours of walking, cycling, doing sports, gardening, doing handiwork/repairs and housework), N = 1,173

## 4.1. Multiple linear regression

Coefficients	Estimate	Std. Error	t value	Pr(>|t|)

(Intercept)	6.0867	3.0440	2.000	0.04578 *

Retirement	4.4696	1.1015	4.058	5.29e-05 ***

Mediterranean diet	1.1049	0.2530	4.368	1.37e-05 ***

Past physical activity behaviour	4.2415	0.6283	6.751	2.32e-11 ***

Female sex	7.4073	1.4155	5.233	1.98e-07 ***

Income: 2001 to 4000 Euro	-3.3612	1.2758	-2.635	0.00853 **

Income: >4000 Euro	-6.6803	1.6365	-4.082	4.77e-05 ***

Income: Not specified	-4.9835	2.1988	-2.266	0.02361 *

Number of people in the household	-1.1018	0.5882	-1.873	0.06132 .

Depression	-0.3076	0.1142	-2.694	0.00717 **

Air pollution	2.0266	0.9822	2.063	0.03931 *

Housing: Two-family-terraced house	-4.0987	1.3560	-3.023	0.00256 **

Housing: Multi-party house with a maximum of 9 units	-1.1330	1.3218	-0.857	0.39153

Housing: Multi-party house with 10 or more units	-1.8627	1.4381	-1.295	0.19551

Housing: Others	-5.3094	8.5251	-0.623	0.53355

Low back pain	0.1253	0.0678	1.848	0.06485 .

Signif. codes: 0 ‘***’ 0.001 ‘**’ 0.01 ‘*’ 0.05 ‘.’ 0.1 ‘ ’ 1

Residual standard error: 16.91 on 1157 degrees of freedom

Multiple R-squared: 0.1666,	Adjusted R-squared: 0.1558

F-statistic: 15.42 on 15 and 1157 DF, p-value: < 2.2e-16

## 4.2. Machine learning model

| Model | MAE | RMSE |
| --- | --- | --- |
| XGBRegressor | 11.26 | 15.70 |
| RandomForestRegressor | 11.71 | 16.42 |
| SVM | 11.98 | 17.09 |
| KNeighborsRegressor | 11.96 | 16.40 |
| DecisionTreeRegressor | 12.61 | 16.77 |
| MAE: mean absolute error  RMSE: root mean squared error  SVM: support vector machines | | |

Top 15 features by XGBRegressor model
