# Supplementary material for: Correlates of physical activity behaviour in a population sample with increased cardiovascular risk and established cardiovascular disease: Cross-sectional analysis of data from the Paracelsus 10,000 prospective cohort study in Salzburg, Austria
Source: PLoS One. 2026 Aug 19;21(8):e0356607. doi: 10.1371/journal.pone.0356607 (PMC13489455; doi:10.1371/journal.pone.0356607)
Supplement: Supplementary file 2 [file pone.0356607.s002.docx]

**S2 Supporting Information. Characteristics of Paracelsus 10.000 participants with increased cardiovascular risk and established cardiovascular disease: sample included in the analysis (n=1173) versus excluded participants (n=3064).** Figures are n (%) unless stated otherwise.

| Characteristics | | All  n=1173 | Excluded  n = 3064 |
| --- | --- | --- | --- |
| Age (years) | Mean (SD) | 60.5 (6.3) | 58.2 (8.7) |
|  | Missing (n, %) | - | - |
| Sex | Male | 952 (81%) | 2114 (69%) |
|  | Female | 221 (19%) | 688 (31%) |
|  | Missing | - | - |
| Education level | Low (ISCED 0-2) | 65 (6%) | 333 (11%) |
|  | Medium (ISCED 3-5) | 694 (59%) | 1854 (61%) |
|  | High (ISCED 6-8) | 402 (34%) | 769 (25%) |
|  | Other | 12 (1%) | 39 (1%) |
|  | Missing | - | 69 (2%) |
| Occupation | Blue-collar | 122 (10%) | 439 (14%) |
|  | White-collar | 858 (73%) | 1631 (53%) |
|  | Self-employed | 193 (17%) | 349 (12%) |
|  | Missing | - | 645 (21%) |
| Retired | Yes | 490 (42%) | 1277 (42%) |
|  | No | 683 (58%) | 1718 (56%) |
|  | Missing | - | 69 (2%) |
| Migration background | First generation | 86 (7%) | 254 (8%) |
|  | Second generation | 24 (2%) | 73 (2%) |
|  | Not first or second generation | 1063 (91%) | 2538 (83%) |
|  | Missing | - | 199 (7%) |
| Religion | Yes | 896 (76%) | 2182 (71%) |
|  | No | 277 (24%) | 813 (27%) |
|  | Missing | - | 69 (2%) |
| Monthly household income after tax (Euros) | ≤2000 | 297 (25%) | 1202 (39%) |
|  | 2001 to 4000 | 582 (50%) | 1140 (38%) |
|  | >4000 | 218 (19%) | 346 (11%) |
|  | Not specified | 76 (6%) | 307 (10%) |
|  | Missing | - | 69 (2%) |
| Marital status | Living with partner | 1060 (90%) | 1936 (63%) |
|  | Separated or no partner | 113 (10%) | 1059 (35%) |
|  | Missing | - | 69 (2%) |
| Number of children | Median (range) | 3 (1, 12) | 3 (1,12) |
|  | Missing (n, %) | - | 69 (2%) |
| Comorbidity | 0 | 647 (55%) | 1632 (53%) |
|  | 1-2 | 452 (39%) | 1209 (40%) |
|  | ≥3 | 74 (6%) | 220 (7%) |
|  | Missing | - | 3 (<1%) |
| BMI | Mean (SD) | 27.5 (4.3) | 27.6 (4.5) |
|  | Missing (n, %) | - | 1 (<1%) |
| Obesity  (BMI > 30 kg/m2) | No | 904 (77%) | 2293 (75%) |
|  | Yes | 269 (23%) | 770 (25%) |
|  | Missing | - | 1 (<1%) |
| WOMAC gonarthrosis score | Mean (SD) | 7.5 (21.5) | 7.3 (23.3) |
|  | Missing (n, %) | - | 69 (2%) |
| WOMAC coxarthrosis score | Mean (SD) | 3.8 (15.9) | 4.9 (20.7) |
|  | Missing (n, %) | - | 69 (2%) |
| ODI score | Mean (SD) | 5.6 (7.8) | 6.1 (9.1) |
|  | Missing (n, %) | - | 378 (12%) |
| BDI score | Mean (SD) | 3.8 (4.7) | 4.9 (6.1) |
|  | Missing (n, %) | - | 69 (2%) |
| ESS score | Mean (SD) | 6.9 (3.4) | 6.7 (3.7) |
|  | Missing (n, %) | - | 69 (2%) |
| BIS-15 score | Mean (SD) | 23.9 (4.0) | 24.8 (4.2) |
|  | Missing (n, %) | - | 69 (2%) |
| Smoking status | Never | 475 (41%) | 1021 (33%) |
|  | Former | 466 (40%) | 1060 (35%) |
|  | Current | 232 (19%) | 914 (30%) |
|  | Missing | - | 69 (2%) |
| AUDIT score | Mean (SD) | 4.3 (3.3) | 4.2 (3.6) |
|  | Missing (n, %) | - | 417 (14%) |
| Past physical activity behaviour, (hours per week of doing sports from at ages 5-19, 20-34, 35-49, and 50+) | Mean (SD) | 3.0 (0.8) | 2.9 (0.9) |
|  | Missing (n, %) | - | 903 (29%) |
| Inventory on Mediterranean diet score | Mean (SD) | 6.3 (2.1) | 6.3 (2.1) |
|  | Missing (n, %) | - | 783 (25%) |
| Number of people in the household | Median (range) | 1 (1, 8) | 1 (1,11) |
|  | Missing (n, %) | - | 950 (31%) |
| Type of housing | Multi-party house with 10 or more units | 223 (19%) | 769 (25%) |
|  | Multi-party house with a maximum of 9 units | 285 (24%) | 826 (27%) |
|  | Two-family terraced house | 256 (22%) | 561 (18%) |
|  | Detached house | 405 (34%) | 815 (27%) |
|  | other | 4 (1%) | 24 (1%) |
|  | Missing | - | 69 (2%) |
| Size of living quarters, (m^2^) | Mean (SD) | 114.4 (46.2) | 100 (51) |
|  | Missing (n, %) | - | 149 (5%) |
| Population density | Dense | 609 (52%) | 1567 (51%) |
|  | Intermediate | 499 (42%) | 1301 (42%) |
|  | Rural | 65 (6%) | 143 (5%) |
|  | Missing | - | 53 (2%) |
| Environmental noise | Mean (SD) | 1.3 (0.5) | 1.3 (0.6) |
|  | Missing (n, %) | - | 335 (11%) |
| Air pollution | Mean (SD) | 1.3 (0.5) | 1.3 (0.5) |
|  | Missing (n, %) | - | 273 (9%) |
| Physical activity, (hours per week) | Walking, cycling & doing sports, mean (SD) | 13.9 (11.6) | 13.4 (10.6) |
|  | Missing (n, %) | - | 805 (26%) |
|  | Gardening, doing  handiwork/ repairs & housework, mean (SD) | 10.8 (11.1) | 11 (10.9) |
|  | Missing (n, %) | - | 704 (23%) |
| Cambridge Activity Index | Active | 446 (38%) | 953 (31%) |
|  | Moderately active | 308 (26%) | 686 (23%) |
|  | Moderately inactive | 341 (29%) | 655 (21%) |
|  | Inactive | 78 (7%) | 279 (9%) |
|  | Missing | - | 491 (16%) |
| Recreational Activity Index | Active | 516 (44%) | 949 (31) |
|  | Moderately active | 268 (23%) | 505 (17%) |
|  | Moderately inactive | 183 (15%) | 377 (12%) |
|  | Inactive | 206 (18%) | 428 (14%) |
|  | Missing | - | 805 (26%) |

AUDIT, Alcohol Use Disorder Identification Test

BDI, Beck Depression Inventory

BIS-15, Barratt Impulsiveness Scale

BMI, Body Mass Index

CVD, cardiovascular disease

ESS, Epworth Sleepiness Scale

ISCED, International Standard Classification of Education

N/A, not applicable

ODI, Oswestry Disability Index

WOMAC, Western Ontario and McMaster Universities Osteoarthritis Index
