# Supplementary material for: Correlates of physical activity behaviour in a population sample with increased cardiovascular risk and established cardiovascular disease: Cross-sectional analysis of data from the Paracelsus 10,000 prospective cohort study in Salzburg, Austria
Source: PLoS One. 2026 Aug 19;21(8):e0356607. doi: 10.1371/journal.pone.0356607 (PMC13489455; doi:10.1371/journal.pone.0356607)
Supplement: Supplementary file 3 [file pone.0356607.s004.docx]

Dear Dr. Nejadghaderi,

On behalf of our group of authors, we thank the journal and the reviewers for their consideration of our manuscript and the helpful comments provided. We have prepared a revised manuscript with track changes and a clean version of the revised manuscript, and we have provided out point-by point responses to journal requirements and reviewer comments below.

Thank you again for considering our work.

Sincerely,

Dr. Stefan Tino Kulnik, MRes FESC	Salzburg, 23^rd^ July 2026

**Journal Requirements**

When submitting your revision, we need you to address these additional requirements.

**AUTHORS:** We thank the editor for their guidance and have addressed these additional journal requirements as listed below.

1. Please ensure that your manuscript meets PLOS ONE's style requirements, including those for file naming. The PLOS ONE style templates can be found at

https://journals.plos.org/plosone/s/file?id=wjVg/PLOSOne_formatting_sample_main_body.pdf and 
https://journals.plos.org/plosone/s/file?id=ba62/PLOSOne_formatting_sample_title_authors_affiliations.pdf.

**AUTHORS:** Thank you, we have adapted the manuscript and file names according to the PLOS ONE style templates.

2. For studies involving third-party data, we encourage authors to share any data specific to their analyses that they can legally distribute. PLOS recognizes, however, that authors may be using third-party data they do not have the rights to share. When third-party data cannot be publicly shared, authors must provide all information necessary for interested researchers to apply to gain access to the data. (https://journals.plos.org/plosone/s/data-availability#loc-acceptable-data-access-restrictions)

For any third-party data that the authors cannot legally distribute, they should include the following information in their Data Availability Statement upon submission:
a) A description of the data set and the third-party source
b) If applicable, verification of permission to use the data set
c) Confirmation of whether the authors received any special privileges in accessing the data that other researchers would not have
d) All necessary contact information others would need to apply to gain access to the data

**AUTHORS:** Thank you for this guidance. We have amended the data availability statement to describe the data set and to include a link to the Paracelsus 10,000 website, which lists contact details for researchers who seek permission to access the data.

3. You have indicated that data is available from [a.bito@salk.at ].  Please can we ask you to provide us with a general contact email address for the data requests, so readers can request access in perpetuity. If a general email is not available please provide a link to a website where readers can obtain access to data.

**AUTHORS:** Thank you for this guidance. There is no generic contact email address provided for data requests, but interested parties are asked to contact the study coordinator ([a.bito@salk.at](mailto:a.bito@salk.at)). There is however a project website that lists these contact details (<https://salk.at/uniklinikum-salzburg/paracelsus-10000>), and instead of the email address we have now provided the link to the website in the data availability statement.

4. Please include captions for your Supporting Information files at the end of your manuscript, and update any in-text citations to match accordingly. Please see our Supporting Information guidelines for more information: http://journals.plos.org/plosone/s/supporting-information.

**AUTHORS:** We have added captions for the supporting information files at the end of the manuscript and have updated the in-text citations accordingly.

5. If the reviewer comments include a recommendation to cite specific previously published works, please review and evaluate these publications to determine whether they are relevant and should be cited. There is no requirement to cite these works unless the editor has indicated otherwise.

**AUTHORS:** Thank you for this guidance. On this occasion, there were no recommendations from reviewers to cite specific previously published works.

**Point-by-point response to reviewers**

**AUTHORS:** We would like to thank both reviewers for their detailed and helpful review of our manuscript. We believe that the reviewers’ feedback has strengthened the manuscript and hope that our revision adequately incorporates and addresses all the reviewers’ comments.

**Reviewer #1**

Thank you for the opportunity to review this manuscript that investigated correlates of physical activity in 1,173 people with increased cardiovascular risk or established CVD. It is important to understand the relationship between certain factors and physical activity to help target physical activity interventions for both primary prevention in this high risk group and secondary prevention of CVD. This manuscript adds to the evidence base of these relationships for both of these populations. Below are some comments that will hopefully improve the manuscript.

**AUTHORS:** We thank the reviewer for their helpful comments on the manuscript, which we have incorporated in the revised version. We hope that our revision and responses adequately address all the reviewer’s points.

Abstract
Background/Aims: Please explain what you mean by ‘heart-healthy’ PA or remove it. Currently it sounds different to the public health PA guidelines that are recommended in clinical guidelines.

**AUTHORS:** We have removed ‘heart-healthy’.

Results: Please write ‘comorbidity’ rather than ‘(co)morbidity’.

**AUTHORS:** We have amended to ‘comorbidity’.

Introduction
Page 4, line 101. I think the word ‘and’ is missing between 2013 and 2020.

**AUTHORS:** Thank you, we have amended ‘and’.

Page 5, line 115. Reference 14 is not a peer reviewed journal, so I would remove this and find a better reference to support your statement.

**AUTHORS:** Reference 14 cites the preprint of our systematic review of factors associated with physical activity behaviour specifically in the cardiac population. Unfortunately, this review is currently still under peer review. To our knowledge, there has been no other published review focusing on factors associated with physical activity behaviour (1) specifically in populations with cardiovascular disease, (2) specifically in everyday life (as opposed to during supervised cardiac rehabilitation provision), and (3) collating observational quantitative association statistics, as we did in our review.

We can, however, support our statement that no such data have been published from the Austrian context by referring to a published umbrella review by Collado-Mateo et al. (2021) that included 55 articles, of which 7 were reviews focusing on cardiovascular conditions. We have replaced reference 14 (now reference 15) with this article by Collado-Mateo et al. and have amended the sentence accordingly:

“Several systematic reviews have focused specifically on this population, yet data from the Austrian context is lacking in this literature (15).”

In the preceding sentence, we have added a new reference (Gutenberg et al., 2022) to support our statement that factors influencing physical activity behaviour in people with cardiovascular disease might differ from the general population.

Line 121. Associations are correlations and can’t determine causation, therefore they can’t ‘predict’. Please change this wording.

**AUTHORS:** We appreciate the reviewer’s comment and have changed to ‘relate to’. In the Methods section (sub-heading ‘Terminology’), we have explicitly stated that conclusions towards a causal relationship cannot be drawn from this cross-sectional observational dataset. We appreciate the reviewer’s pointers to make our language unambiguous throughout the manuscript.

Methods
Line 126. Please provide a brief summary of the P10 study inclusion criteria and whether participants were followed over time (cohort) or recruited at different time points (cross-sectional). Please also provide detail on how data was collected ie: via post, phone, in-person.

**AUTHORS:** We appreciate the author’s suggestion and have added the following passage at the beginning of the Methods:

“In brief, 60,000 people aged 40 to 70 years and living in the city of Salzburg and its surroundings were randomly identified from the population register and invited by letter to participate in the study. Recruitment was stratified, aiming to include an equal number of men and women and 25%, 50% and 25% of participants in age groups 40 to 49 years, 50 to 59 years, and 60 to 70 years, respectively. Recruitment took place from 01 April 2013 until 31 March 2020, at which point 10,044 participants had been included in the study. Each study participant completed data collection in a single study visit on site at the University Hospital Salzburg in the presence of trained study personnel. Medical history, family history and medication were assessed by a structured audio-recorded face-to-face interview. Study personnel specifically questioned participants whether each stated medical condition had been formally diagnosed by a medical doctor. All self-reported questionnaires were completed by participants on a computer in the presence of study personnel who assisted as needed. Since autumn 2020, all study participants have been invited to return for their follow-up visit seven years after their initial study visit (7). While the P10 study is a prospective cohort study, the data analysed for this article represents a cross-sectional dataset collected at the initial study visit.”

Page 6, line 144. Can you please provide some detail about the SCORE2 such as what risk factors this is based on and whether these risk factors self reported?

**AUTHORS:** We thank the author for this suggestion, which we appreciate will be helpful for readers who are less familiar with the SCORE2 and SCORE2-OP risk prediction models. We have added the following sentence:

“The SCORE2 and SCORE2-OP risk models are based on country of residence, age, sex, systolic blood pressure, blood cholesterol (high density lipoprotein and total cholesterol) and current smoking (19,20).”

Of these parameters, only current smoking is usually self-reported, although it could in theory also be assessed objectively by cotinine testing or testing of exhaled carbon monoxide.

Line 153. Was diagnoses self-reported? If so, please make this clear and comment on whether the self-reporting of diagnoses is reliable and valid, if this is the case.

**AUTHORS:** Thank you for raising this point. Medical diagnoses were self-reported by participants in a structured audio-recorded face-to-face interview administered by trained study personnel. Study personnel specifically questioned participants whether each stated medical condition had been formally diagnosed by a medical doctor. We have added this information to the first paragraph of the Methods section.

With regard to the reliability and validity of the self-reporting of diagnoses, we acknowledge the possibility of inaccuracies, e.g., due to recall bias. Capturing diagnoses by official medical records was not considered a feasible option due to disconnected medical documentation systems in Austria (i.e., at the time, there was no established and reliable central electronic patient record).

As a measure to strengthen the reliability and validity of self-reported diagnoses, trained study personnel went through a structured medical history questionnaire together with participants. This questionnaire listed 75 different medical diagnoses. The diagnoses were worded in everyday language rather than medical jargon (e.g., “high blood pressure” rather than “arterial hypertension”, etc.). For each group of medical diagnoses, the question was: “Have you ever been diagnosed with one of the following … (e.g., cardiovascular diseases)?”, followed by the specific diagnoses. Answer options were yes and no. When the participant answered yes for one diagnosis, they were asked their age at the time of diagnosis, and whether they were currently being treated for this condition (yes/no).

This approach is more robust than simply asking an open question, because the list of diagnoses can jog the participant’s memory and therefore reduce recall bias. Additionally, study personnel were trained to specifically ask whether self-reported medical conditions had been formally diagnosed by a medical doctor, and to cross-check with self-reported medication.

Page 7, line 169. Is it possible to include the survey questions in a supplementary file or include a link to the survey if available on a website? It would be useful to see the response options for each question.

**AUTHORS:** We appreciate the reviewers’ suggestion. Unfortunately, the survey is not publicly available, and we do not have permission to reproduce study materials as part of this article.

For the reviewer’s information, most of the data/variables used in our analysis were collected through standard validated questionnaires and patient-reported outcome measures, which we have listed and referenced in the Methods section under the sub-heading “Correlates of physical activity behaviour”.

The wording, format and response options of those questions that captured socio-demographic characteristics and smoking behaviour were taken from a well-established and widely used screening questionnaire of the Austrian Social Insurance.

The questions on environmental noise and air pollution were adapted from environmental medicine questionnaires and were as follows:

“At home, do you currently feel disturbed by noise?” with 4 response options (not at all, hardly, moderately, strongly) for each of the following causes of noise: street noise, railway noise, flight noise, business/industrial noise, noise caused by neighbours.

and

“At home, do you currently feel disturbed by air pollution?” with 4 response options (not at all, hardly, moderately, strongly) for each of the following causes of air pollution: street traffic, households burning fossil fuels, flight traffic, industry/business.

Line 169-171. Please provide references for this sentence referring to research on potential relationships between factors and physical activity.

**AUTHORS:** Thank you, we have added the references.

Lines 179-196. Please include whether these questionnaires included in the P10 were reliable and valid in the population you were looking at (high risk of CVD event, CVD diagnoses) or the general population.

**AUTHORS:** Thank you, we have added the following sentence:

“These standard questionnaires are considered valid and reliable for the general adult population.”

Page 8, lines 199-212. Do any of these physical activity questions ask about intensity ie: MVPA? If not, please make this clear.

**AUTHORS:** Thank you for pointing this out, and we have added the following sentence to clarify:

“Of note, physical activity intensity was not captured, i.e., these data represent the sum of physical activity at light, moderate and vigorous intensity levels.”

Page 10, line 254. Please remove bold letters for ‘MASS’.

**AUTHORS:** We have removed the bold formatting.

Results
Page 11, line 284. Are these characteristics for included participants? If so, please make this clear.

**AUTHORS:** Thank you, these characteristics refer to the participants included in the analysis. We have clarified this in the text.

Lines 28-287. Please include the total n for this subgroup in this sentence and in Figure 1.

**AUTHORS:** Thank you, we have added the total n for this subgroup in the text and in Figure 1.

Lines 287-289. Please include the total n for this subgroup in this sentence and in Figure 1.

**AUTHORS:** Thank you, we have added the total n for this subgroup in the text and in Figure 1.

Table 1. This table is very long. I suggest for dichotomous outcomes, only report one outcome. For other outcomes, possibly only report the most prevalent outcome.

**AUTHORS:** We appreciate the reviewer’s comment. We have included this detailed table to present the data to its fullest extent, describing all variables under investigation for the entire sample and for each of the sub-groups. We would prefer not to remove data categories from the table purely for the purpose of shortening it, because we feel it would make the table more difficult to read and cross-reference with the other tables, and might cause confusion for readers. If the reviewer agrees, we would ask for editorial guidance whether this table can remain in the main manuscript, or whether it should better be moved from the main manuscript to an online supplementary file.

In table 1, the past physical activity reported is much lower than the current physical activity, assuming both are reported a hours per week?

**AUTHORS:** We thank the reviewer for this astute observation. It is correct that both past and current physical activity are reported in hours per week. The much lower reported past physical activity is explained by the fact that the question that was asked to capture this specifically asked about “doing sports in the past”. This therefore excludes the other types of physical activity behaviour that were also captured in the questions about current physical activity (i.e., walking, cycling for transportation, etc.).

We have amended the wording in the manuscript to clarify that past physical activity in fact refers to “doing sports in the past”. In the Method section we have re-worded to “Past physical activity behaviour was recorded from self-reported average hours per week of doing sports at ages 5-19, 20-34, 35-49, and 50+ years.” and in the tables, we have amended to clarify that past physical activity refers to doing sports.

Page 16, lines 299-301. Please include the direction of these variable eg: higher past physical activity, if retired, adherence to the Mediterranean diet, and ? increased air pollution.

**AUTHORS:** Thank you, we have amended as suggested.

Line 301-304. The same applies to this sentence. Sometimes the direction is included but not consistently ie: is it a higher number of people living in the household?

**AUTHORS:** Thank you, we have amended as suggested. It is correct that a higher number of people living in the household was inversely associated with physical activity.

Table 2. Please include n (%) in the third column to make this easier to interpret for the reader.

**AUTHORS:** Thank you, we have inserted n (%) in the third column of Table 2.

Table 3. Why is the p-value for BMI and environmental noise in bold as these p-values at not significant? Please also write p<0.001 in the last line of the table instead of including the exponential value.

**AUTHORS:** We thank the reviewer for pointing out the bold p-values for BMI and environmental noise. This was taken over by mistake from the statistical programme output, because the statistical programme by default highlights p-values below 0.1. As we use 0.05 as the threshold for statistical significance in our analysis, the reviewer rightly points out that this is inconsistent, and we have removed the bold formatting.

In the last line of the table, we have amended to p<0.001.

Discussion
Page 28, Lines 369-380. Please highlight the gender findings here for gardening etc and total physical activity as you include this as a sub-heading later on.

**AUTHORS:** We thank the reviewer for this suggestion and have added the following sentence to the first paragraph of the Discussion section:

“Sex was not associated with the primary physical activity outcome (walking, cycling and doing sports), but female sex was associated with more physical activity for both total physical activity and gardening, doing handiwork/repairs and housework, whereby the association in total physical activity was driven by the difference between sexes in gardening, doing handiwork/repairs and housework.”

Lines 382-388. I think this paragraph would fit better under the ‘Retirement’ sub-heading.

**AUTHORS:** Thank you, we have moved the paragraph to the ‘Retirement’ sub-heading.

Page 29, line 411. I suggest ‘Gender’ should be the fourth heading as the first three factors were more prominent in your findings.

**AUTHORS:** Thank you, we have re-ordered so that ‘Gender’ is now the fourth heading.

Lines 415-417. In the scoping review men were more physically active than women but did this also apply for gardening, etc and total physical activity or only for walking?

**AUTHORS:** Thank you for this question. The finding we cite here from the review by Lee and Collins (2021) in patients after cardiac surgery is based on three primary studies included in the review. Each of these primary studies used a different approach to capture physical activity, but all were self-reported:

The first study (Gortner and Jenkins 1990, reference 27 in the review) asked participants about four physical activity behaviours: walking, lifting, climbing and “general activity”.

The second study (Jenkins and Gortner 1998, reference 28 in the review) asked patients about 16 different walking activities, ranging from “walking from the bed to the bathroom” to “walking four miles”.

The third study (Treat-Jacobson and Lindquist 2004, reference 31 in the review) used the College Alumni Physical Activity Questionnaire to capture number of blocks walked per day, the number of flights of stairs climbed per day, and sports (vigorous or light) in which the participants participated (in hours per week). These responses were then converted to a Physical Activity Index, based on assigned metabolic equivalents of task.

In summary, the finding from the review refers mainly to walking as a way to get around (including stairs climbing), in the first study it also incorporated unspecified “general” physical activities, and in the third study it also incorporated sports. It is possible that gardening might have been included under “general activity” in the first study, but the authors do not comment on that in the article.

Page 31, lines 459-466. Also consider that assessment of physical activity should be broad, including activities such as gardening and household chores/work and total physical activity, particularly for females as your analysis shows that increased activity in these areas is associated with being female.

**AUTHORS:** Thank you for suggesting this point, which we fully agree with and have added to this passage:

“With respect to the assessment of physical activity, our findings highlight the importance of capturing a broad range of physical activity behaviours, including gardening and household work, particularly for women, as our analysis shows that increased activities in these areas was associated with female sex.”

Page 32, lines 482-483. Please include in the Methods that the EPIC physical activity questionnaire has been validated.

**AUTHORS:** Thank you for this comment, we have included in the Methods (sub-section “Outcome variables”) that the EPIC questionnaire has been validated:

“This questionnaire has been validated in large European cohorts (31,32) and captures not only…”

Lines 488-489. Can you please add the sample size calculation for this analyses to the Methods?

**AUTHORS:** Thank you for this suggestion. As this was a secondary analysis of an existing dataset, no formal a priori sample size calculation was performed. We have now added a justification that the final sample size of 1,173 participants was considered adequate for the planned multiple regression analyses relative to the number of candidate variables included in the regression models, and is consistent with commonly cited heuristic sample size rules for multiple regression, as reviewed by Green (1991).

Page 33, line 506. I think comorbidity and depression were both inversely associated with physical activity behavior. If this is correct, please include depression in this sentence.

**AUTHORS:** Thank you for pointing this out, we have included depression in this sentence.

Page 39. Reference 55 is the same as referenced 6. Please remove this duplicate.

**AUTHORS:** Thank you for spotting this duplicate reference and apologies for our oversight. We have corrected the reference.

Figure 1. Is it possible to improve the quality of this image as it is quite blurry?

**AUTHORS:** Thank you for pointing this out, and we apologise for the low quality of Figure 1. We have replaced it with a file at 600 dpi resolution.

**Reviewer #2**

Thank you for the opportunity to review this manuscript by Kulnik et al. The study addresses an important topic “Correlates of physical activity behaviour in a population sample with increased cardiovascular risk and established cardiovascular disease: data from the Paracelsus 10,000 prospective cohort study in Salzburg, Austria”. The use of data from the Paracelsus 10,000 study is a strength, and the topic is relevant for cardiovascular prevention. However, there are several concerns about the study design, statistical analysis, and interpretation require clarification before the findings can be appropriately interpreted.

**AUTHORS:** We thank the reviewer for their helpful comments on the manuscript, which we have incorporated in the revised version. We hope that our revision and responses adequately address all the reviewer’s points.

Major comments

1) Methods: The manuscript repeatedly refers to the Paracelsus 10,000 study as a prospective cohort, which is accurate for the parent study. However, the present analysis appears to be a cross-sectional analysis of data from this cohort. This should be stated more explicitly throughout the manuscript, including in the title, abstract, methods, and discussion. I suggest revising the title to indicate that this is a “cross-sectional analysis of data from the Paracelsus 10,000 prospective cohort study” or similar. This clarification is important because the current wording may give readers the impression that the analysis itself is longitudinal or prospective.

**AUTHORS:** We thank the reviewer for this important clarification, which we fully agree with. We have amended the title as suggested by the reviewer and have revised the wording in the abstract, methods and discussion to clarify that this dataset is cross-sectional and not longitudinal or prospective. For example, in the first paragraph of the Methods section, we have added the following sentence:

“While the P10 study is a prospective cohort study, the data analysed for this article represents a cross-sectional dataset collected at the first P10 study visit.”

2) Methods: The manuscript combines bivariate analyses, multivariable regression models, and machine-learning approaches. This is acceptable in principle, but these methods answer different questions and should be interpreted separately. Bivariate analyses describe crude associations, regression models estimate adjusted associations, and machine-learning models assess prediction or feature importance. These approaches should not be presented as equivalent forms of evidence for the same conclusion.

**AUTHORS:** We completely agree with the reviewer's perspective. These analytical methods address distinct research questions and evaluate different dimensions of the data; therefore, they should not be treated as equivalent forms of evidence for a single conclusion. We have revised our manuscript to ensure this clear conceptual separation. In the introductory paragraph to the “Statistical analysis” section, we have

1. **clarified the distinct roles:** we now explicitly state that these three approaches answer distinct research questions (crude associations vs. adjusted inference vs. predictive importance) and are interpreted independently rather than being presented as equivalent or identical evidence.
2. **corrected the analytical objectives:** we removed the statement that the machine learning models "served to corroborate" the regression models. Instead, we now state that the final sets of correlates selected by the regression models are evaluated *alongside* the predictive feature importances from the ML models to provide *complementary insights* into physical activity behaviors from different statistical perspectives.

3) Methods: The use of bidirectional stepwise regression based on AIC also requires clearer justification. Stepwise procedures can produce unstable models, particularly when candidate predictors are correlated. If the aim is explanatory, a prespecified model based on prior evidence or a conceptual framework would be preferable. If the aim is prediction or variable selection, penalized regression approaches such as LASSO, ridge regression, or elastic net could be considered as alternatives or sensitivity analyses. LASSO can support sparse variable selection, ridge regression can help address multicollinearity, and elastic net may be useful when groups of predictors are correlated. These models could be tuned using cross-validation and compared with the reported machine-learning models.

**AUTHORS:** We agree that stepwise regression has well-recognised limitations, including potential model instability and biased coefficient estimates. Our objective, however, was not to develop an optimal prediction model but to identify a parsimonious set of correlates associated with physical activity within an exploratory analysis. For this reason, we chose bidirectional stepwise selection based on AIC, which represents a commonly used model selection approach balancing model fit and complexity. We have clarified this rationale in the Discussion.

4) Methods: The distinction between “regression” and “machine learning” should also be clarified, since regression models can also be considered supervised statistical-learning models. The more relevant distinction is between interpretable regression models for adjusted associations and more flexible prediction-oriented models such as random forests or gradient boosting.

**AUTHORS:** We thank the reviewer for this excellent and clarifying recommendation. We completely agree that classical regression models fall under the umbrella of supervised statistical learning, and that the crucial distinction lies in their assumptions and utility. We have updated our manuscript as follows to address this point:

1. **Updated the terminology and headings:** we now explicitly distinguish between interpretable, parametric regression models used to estimate adjusted associations (inference) and flexible, non-parametric algorithms (e.g., Random Forest, KNN, XGBoost) used to assess predictive performance and feature importance. The main subheadings have been updated to reflect this:
   *- “Parametric regression analysis”*
   *- “Non-parametric machine learning analysis”*
2. **Clarified the rationale in the text:** we added explicit text explaining that the parametric models were chosen to provide clear, confounding-adjusted effect estimates for hypothesis testing, while the non-parametric machine learning models were deployed to evaluate global predictive capabilities and capture complex, non-linear interactions without assuming a rigid functional form.

5) Methods: The manuscript includes sociodemographic, clinical, psychological/cognitive, lifestyle, and environmental variables as candidate correlates of physical activity behaviour. However, these variable groups differ substantially in their temporal and causal interpretation. Clinical variables such as CVD risk, comorbidity, and body mass index may be causes of lower physical activity, consequences of prior physical activity, mediators, or bidirectionally related to physical activity. This issue is particularly important in a cross-sectional analysis. For example, higher BMI or musculoskeletal pain may reduce current physical activity, but long-term low physical activity may also contribute to these clinical conditions. The authors should therefore avoid presenting these variables as determinants or intervention targets unless temporal ordering can be supported. A clearer conceptual framework, such as a table or directed acyclic graph classifying variables as potential confounders, exposures, mediators, or consequences, would improve interpretation.

**AUTHORS:** We thank the reviewer for this comment. We fully agree that our dataset and analysis present inherent limitations when it comes to interpreting causality or mechanisms of action. In the Methods section (sub-heading ‘Terminology’), we have explicitly stated that conclusions towards a causal relationship cannot be drawn from this cross-sectional observational dataset, and in response to this comment and also a comment from reviewer 1, we have carefully reviewed the manuscript and amended the wording throughout to be consistent and unambiguous in this respect. We hope that by making these amendments, we now present a coherently cautious and transparent interpretation of our work.

With regard to a conceptual framework or a hypothesised pathway of action, we fully agree with the reviewer that it is a possible and valuable approach to develop such a framework or model *a priori*, e.g., from theory or prior evidence, that is then tested / verified based on this new dataset. However, we deliberately decided on a more open and exploratory approach to utilise and evaluate as many variables as possible from the P10 dataset, because this provided an opportunity to also obtain an analytic signal about variables that are much less commonly seen in this literature (such as religion, housing circumstances, and environmental conditions).

The role of “CVD risk” also requires clarification. It appears to be used both as part of the sample definition or subgroup classification and as a clinical correlate. The authors should specify exactly how this variable was included in the regression and machine-learning models, and whether it represents SCORE2/SCORE2-OP category, established CVD status, or the combined primary/secondary prevention grouping. Possible overlap with variables such as age, sex, smoking, BMI, and comorbidity should also be addressed.

**AUTHORS:** We appreciate the reviewer’s comment. The reviewer is correct in that the “CVD risk” characteristic / variable was used for two separate purposes.

In the first instance, we needed to identify those with CVD risk out of all 10,044 P10 study participants. For this we conceptualised CVD risk according to the common clinical distinction into primary and secondary CVD prevention.

Primary prevention in this context refers to individuals who have an elevated cardiovascular risk, but do not have an established clinical diagnosis of CVD. To identify elevated CVD risk, we used the validated SCORE2/SCORE2-OP risk model and included all individuals with high or very high risk according to SCORE2/SCORE2-OP.

Secondary prevention in this context refers to individuals who have an established clinical diagnosis of CVD, which usually means that they will have suffered significant cardiovascular symptoms that have brought their underlying CVD to the fore. For our study, we classified all individuals with one of the following clinical diagnoses to the secondary prevention sub-sample: coronary heart disease, chronic heart failure, peripheral arterial disease, abdominal aortic aneurysm, atrial fibrillation and stroke. This is explained in the manuscript in the Methods section under the sub-heading “Study population”.

Secondly, we also used the same CVD risk classification as a potential correlate of physical activity in our analyses. The 3 CVD risk categories were – as above – primary prevention with high risk, primary prevention with very high risk, and secondary prevention. In this order, the 3 risk categories present a hierarchical ordering from the least to the most severe clinical presentation.

We apologise for a mistake in Tables 1 and 2, where for the primary prevention CVD risk categories we mistakenly wrote “medium risk” instead of “high risk”, and “high risk” instead of “very high risk”. This has been corrected.

We realise that in the manuscript we did not describe in detail how we used the CVD risk variable as a potential correlate of physical activity in the analyses and apologise for this. We have added the following passage in the Methods section under the sub-heading “Correlates of physical activity behaviour”:

“CVD risk was defined as described above according to the SCORE2/SCORE2-OP risk categories and established diagnosis of CVD. In order from least to most severe CVD risk, the categories are: primary prevention sub-group with high risk, primary prevention sub-group with very high risk, and secondary prevention sub-group.”

We appreciate the reviewer’s comment about possible overlap between CVD risk and variables such as age, sex, smoking, BMI, and comorbidity. The reviewer rightly points out that there is some inherent overlap with age, sex and smoking in the primary prevention sub-group, as these 3 variables are incorporated in the SCORE2/SCORE2-OP risk model; and some inherent overlap with comorbidity in the secondary prevention sub-group, as an established clinical diagnosis of CVD will be reflected in the comorbidity variable also. In the regression analysis, we checked the variance inflation factors for the full regression models (before stepwise selection), and they were all below 2, suggesting that multicollinearity was not a concern. We have added the following sentence to the Methods section under the sub-heading “Regression analysis”:

“Variance inflation factors were examined for the full regression models (before stepwise selection) and were consistently below 2, suggesting that multicollinearity was not a concern.”

6) Results: Because the analysis is cross-sectional, the results should not be interpreted as evidence that the identified variables cause or determine physical activity behaviour. Terms such as “predict,” “influence,” and implications for personalised physical-activity promotion should be used cautiously unless the authors clearly mean statistical prediction rather than temporal or causal prediction.

**AUTHORS:** We appreciate the reviewer’s comment and caution. We fully agree that our dataset and analysis present inherent limitations when it comes to interpreting causality or mechanisms of action. In the Methods section (sub-heading ‘Terminology’), we have explicitly stated that conclusions towards a causal relationship cannot be drawn from this cross-sectional observational dataset, and in response to this comment and also a comment from reviewer 1, we have carefully reviewed the manuscript and amended the wording throughout to be consistent and unambiguous in this respect. We hope that by making these amendments, we now present a coherently cautious and transparent interpretation of our work.

7) The Results section should more clearly distinguish findings from bivariate analyses, multivariable regression models, and machine-learning analyses. Please specify which findings apply to the primary outcome and which apply to secondary outcomes, and indicate whether each finding is model-specific or consistent across analytical approaches. Also, effect estimates should be reported and interpreted more consistently. The relatively low explained variance of the primary regression model should also be acknowledged, as it indicates that the identified variables explain only a limited proportion of variability in physical activity behaviour.

**AUTHORS:** We thank the reviewer for raising these points. We view the bivariate analysis as a preliminary analysis, which is included in the manuscript for completeness and transparency according to the STROBE reporting guideline. Our interpretation focuses on the multiple linear regression and complementary machine learning analyses.

In the Results section, following the description of sample characteristics, we present the results of all three analyses (bivariate, multiple linear regression, machine learning) for the primary outcome under the sub-heading “Primary physical activity outcome”. Here, we highlight statistically significant correlates in the multiple linear regression; and we focus on the feature performance of the top-15 features of the XGBRegressor model, as all five machine learning models had similar performance, but the XGBRegressor had slightly superior performance statistics.

With regard to the secondary outcomes, we present a high-level summary in the main text under the sub-heading “Secondary physical activity outcomes”, because these results concern four further outcomes (CAI; RAI; weekly hours of gardening, doing handiwork/repairs and housework; and weekly hours of total physical activity), and it would take up a lot of space in the manuscript to report these in detail. For completeness and transparency, we present the detailed results of all these analyses in the supporting information file S1. In the main text, we highlight similarities and differences between the primary and secondary physical activity outcomes according to the multiple regression models.

The reviewer is absolutely right to point out the low explained variance of the primary regression model, which we also state in the Results section. In the limitations section, we write:

“We acknowledge that this analysis was reliant on the variables available within the P10 dataset. The dataset lacked a number of correlates of physical activity that are well-documented in the literature, including individual cognitive/psychological factors such as self-efficacy and intention [6,57], interpersonal factors such as social support and loneliness [52,58] and environmental factors such as the built environment [59]. We suggest that this accounts for the large proportion of the outcome variation that was not explained by our analysis models.”

8) Results: For the machine-learning analyses, SHAP values should be interpreted as contributions to model prediction, not as evidence of causal or clinical importance. If model performance was similar across algorithms, conclusions based on the single best-performing model should be presented cautiously.

**AUTHORS:** We thank the reviewer for highlighting this important point and have added the following sentence at the end of the sub-section “Non-parametric machine learning analysis” in the Methods:

“Importantly, SHAP values should be interpreted as contributions to model prediction, not as evidence of causal or clinical importance.”

9) The Discussion should more clearly state that the findings represent cross-sectional associations, not determinants of physical activity behaviour. Statements about prevention, tailoring, or personalised physical-activity promotion should be softened unless supported by longitudinal or interventional evidence. Finally, the possibility of reverse causation should be discussed, particularly for clinical variables such as BMI, pain, comorbidity, and depression, which may both influence and be influenced by physical activity.

**AUTHORS:** We appreciate the author’s comment and suggestion and have added the following passage at a prominent position in the Discussion (second paragraph of the Discussion):

“When interpreting these findings, it is important to bear in mind the limitations inherent to a cross-sectional dataset. These findings represent correlations, not determinants, of physical activity behaviour and serve an exploratory or hypothesis-generating purpose. Longitudinal or interventional evidence is required to demonstrate causal relationships. Moreover, in a cross-sectional dataset the possibility of reverse causation needs to be considered, particularly for clinical variables such as body mass index, pain, comorbidity and depression, which may both influence and be influenced by physical activity.”

Minor comments

10) The Abstract should be revised for clarity and precision. I think, the total size of the original Paracelsus 10,000 cohort does not need to be reported in the Abstract; the focus should be on the analytical sample used in the present study. I suggest moving the analytical sample size, mean age, and female proportion to the first sentence of the Results section.

**AUTHORS:** Thank you for these suggestions, which we have incorporated in the revised abstract.

The phrase “greater (co)morbidity” is vague and should be specified. Please indicate which comorbidity variable or specific comorbidities were associated with physical activity behaviour.

**AUTHORS:** We thank the reviewer for this comment. In the Methods section under the sub-heading “Correlates of physical activity” we describe how we operationalised the variable “comorbidity”:

(In response to a comment from reviewer #1, we have changed “(co)morbidity” to “comorbidity” throughout the manuscript.)

“Comorbidity was categorised in 0, 1-2 or ≥3 of the following diagnoses: type 2 diabetes, type 1 diabetes, coronary heart disease, chronic heart failure, peripheral arterial disease, rheumatoid arthritis, cirrhosis of the liver, chronic hepatitis, gastric or duodenal ulcer, chronic obstructive pulmonary disease, stroke, Alzheimer’s disease or other type of dementia, chronic kidney failure, pancreatic cancer, bladder cancer, lung cancer, breast cancer, brain tumour and ovarian cancer.”

We initially intended to use the Charlson Comorbidity Index; however, because of the way in which participants’ medical history was collected, certain aspects that are required for calculating the Charlson Comorbidity Index were not available.

The Methods section of the Abstract states that stepwise multiple regression and machine-learning models were used, but the specific machine-learning model(s) should be briefly indicated. The Results section should also clarify which model produced the reported effect estimates and what metric these estimates represent, for example adjusted regression coefficients for weekly hours of walking, cycling, and sports. At present, the Abstract results are somewhat difficult to interpret.

**AUTHORS:** Thank you, we have added the five machine learning approaches in the abstract, and we have clarified that the adjusted effect estimates on the primary PA outcome (specified in the Methods section of the abstract as “weekly hours of waling, cycling and doing sports”) were derived from the stepwise multiple regression.

11) Methods: the bivariate analysis section should explain how the authors chose between Pearson’s and Spearman’s correlation coefficients. Please specify whether Pearson’s correlation was used for approximately linear relationships between continuous variables, and whether Spearman’s correlation was used for ordinal variables, skewed distributions, outliers, or monotonic but non-linear relationships. Or how the normality of continuous variables were assessed.

**AUTHORS:** We thank the reviewer for this suggestion. We have clarified in the Methods that Pearson’s correlation coefficient was used for approximately linear associations between continuous variables with approximately symmetric distributions, whereas Spearman’s rank correlation coefficient was used for ordinal variables.

12) Discussion-Limitation: The large reduction from the eligible cohort to the complete-case analytical sample should be more clearly acknowledged as a potential source of selection bias. If participants with complete data differed from excluded participants in characteristics related to physical activity, the reported associations may not be fully generalizable to the broader population with increased cardiovascular risk or established cardiovascular disease.

**AUTHORS:** We appreciate the reviewer’s point. For transparency, we present the participant characteristics of the excluded group in an online supplemental file (S2). In comparison with the included group, there are discrepancies which we describe in the manuscript: the excluded group had a higher proportion of women (31% versus 19%) and higher proportions of participants with the lowest education level (11% versus 6%) and the lowest income category (39% versus 25%).

We have added the following statement to acknowledge these differences as a potential source of selection bias: “These differences are acknowledged as a potential source of selection bias.”
