# Supplementary material for: Correlates of physical activity behaviour in a population sample with increased cardiovascular risk and established cardiovascular disease: Cross-sectional analysis of data from the Paracelsus 10,000 prospective cohort study in Salzburg, Austria
Source: PLoS One. 2026 Aug 19;21(8):e0356607. doi: 10.1371/journal.pone.0356607 (PMC13489455; doi:10.1371/journal.pone.0356607)
Supplement: Peer review history

# Peer review: Decision Letter 0

**DOI:** 10.1371/journal.pone.0356607.r001

**Authors:** Seyed Aria Nejadghaderi

17 Jun 2026

Dear Dr. Kulnik,

Thank you for submitting your manuscript to PLOS ONE. After careful consideration, we feel that it has merit but does not fully meet PLOS ONE’s publication criteria as it currently stands. Therefore, we invite you to submit a revised version of the manuscript that addresses the points raised during the review process.

Please submit your revised manuscript by Aug 01 2026 11:59PM. If you will need more time than this to complete your revisions, please reply to this message or contact the journal office at plosone@plos.org. When you're ready to submit your revision, log on to https://www.editorialmanager.com/pone/ and select the 'Submissions Needing Revision' folder to locate your manuscript file.

- A letter that responds to each point raised by the academic editor and reviewer(s). You should upload this letter as a separate file labeled 'Response to Reviewers'.
- A marked-up copy of your manuscript that highlights changes made to the original version. You should upload this as a separate file labeled 'Revised Manuscript with Track Changes'.
- An unmarked version of your revised paper without tracked changes. You should upload this as a separate file labeled 'Manuscript'.

If you would like to make changes to your financial disclosure, please include your updated statement in your cover letter. Guidelines for resubmitting your figure files are available below the reviewer comments at the end of this letter.

If applicable, we recommend that you deposit your laboratory protocols in protocols.io to enhance the reproducibility of your results. Protocols.io assigns your protocol its own identifier (DOI) so that it can be cited independently in the future. For instructions see: https://journals.plos.org/plosone/s/submission-guidelines#loc-laboratory-protocols. Additionally, PLOS ONE offers an option for publishing peer-reviewed Lab Protocol articles, which describe protocols hosted on protocols.io. Read more information on sharing protocols at https://plos.org/protocols?utm_medium=editorial-email&utm_source=authorletters&utm_campaign=protocols.

As the corresponding author, your ORCID iD is verified in the submission system and will appear in the published article. PLOS supports the use of ORCID, and we encourage all coauthors to register for an ORCID iD and use it as well. Please encourage your coauthors to verify their ORCID iD within the submission system before final acceptance, as unverified ORCID iDs will not appear in the published article. *Only*  the individual author can complete the verification step; PLOS staff *cannot*  verify ORCID iDs on behalf of authors.

We look forward to receiving your revised manuscript.

Kind regards,

Seyed Aria Nejadghaderi

Academic Editor

PLOS One

Journal Requirements:

When submitting your revision, we need you to address these additional requirements.

1. Please ensure that your manuscript meets PLOS ONE's style requirements, including those for file naming. The PLOS ONE style templates can be found at

https://journals.plos.org/plosone/s/file?id=wjVg/PLOSOne_formatting_sample_main_body.pdf and

https://journals.plos.org/plosone/s/file?id=ba62/PLOSOne_formatting_sample_title_authors_affiliations.pdf.

2. For studies involving third-party data, we encourage authors to share any data specific to their analyses that they can legally distribute. PLOS recognizes, however, that authors may be using third-party data they do not have the rights to share. When third-party data cannot be publicly shared, authors must provide all information necessary for interested researchers to apply to gain access to the data. (https://journals.plos.org/plosone/s/data-availability#loc-acceptable-data-access-restrictions)

For any third-party data that the authors cannot legally distribute, they should include the following information in their Data Availability Statement upon submission:

a) A description of the data set and the third-party source

b) If applicable, verification of permission to use the data set

c) Confirmation of whether the authors received any special privileges in accessing the data that other researchers would not have

d) All necessary contact information others would need to apply to gain access to the data

3. You have indicated that data is available from [a.bito@salk.at ].  Please can we ask you to provide us with a general contact email address for the data requests, so readers can request access in perpetuity. If a general email is not available please provide a link to a website where readers can obtain access to data.

4. Please include captions for your Supporting Information files at the end of your manuscript, and update any in-text citations to match accordingly. Please see our Supporting Information guidelines for more information: http://journals.plos.org/plosone/s/supporting-information.

5. If the reviewer comments include a recommendation to cite specific previously published works, please review and evaluate these publications to determine whether they are relevant and should be cited. There is no requirement to cite these works unless the editor has indicated otherwise.

[Note: HTML markup is below. Please do not edit.]

Reviewers' comments:

Reviewer's Responses to Questions

**Comments to the Author**

1. Is the manuscript technically sound, and do the data support the conclusions?

Reviewer #1: Yes

Reviewer #2: Partly

**********

2. Has the statistical analysis been performed appropriately and rigorously?

Reviewer #1: Yes

Reviewer #2: No

**********

3. Have the authors made all data underlying the findings in their manuscript fully available?

The PLOS Data policy

Reviewer #1: No

Reviewer #2: No

**********

4. Is the manuscript presented in an intelligible fashion and written in standard English?

Reviewer #1: Yes

Reviewer #2: Yes

**********

Reviewer #1: Thank you for the opportunity to review this manuscript that investigated correlates of physical activity in 1,173 people with increased cardiovascular risk or established CVD. It is important to understand the relationship between certain factors and physical activity to help target physical activity interventions for both primary prevention in this high risk group and secondary prevention of CVD. This manuscript adds to the evidence base of these relationships for both of these populations. Below are some comments that will hopefully improve the manuscript.

Abstract

Background/Aims: Please explain what you mean by ‘heart-healthy’ PA or remove it. Currently it sounds different to the public health PA guidelines that are recommended in clinical guidelines.

Results: Please write ‘comorbidity’ rather than ‘(co)morbidity’.

Introduction

Page 4, line 101. I think the word ‘and’ is missing between 2013 and 2020.

Page 5, line 115. Reference 14 is not a peer reviewed journal, so I would remove this and find a better reference to support your statement.

Line 121. Associations are correlations and can’t determine causation, therefore they can’t ‘predict’. Please change this wording.

Methods

Line 126. Please provide a brief summary of the P10 study inclusion criteria and whether participants were followed over time (cohort) or recruited at different time points (cross-sectional). Please also provide detail on how data was collected ie: via post, phone, in-person.

Page 6, line 144. Can you please provide some detail about the SCORE2 such as what risk factors this is based on and whether these risk factors self reported?

Line 153. Was diagnoses self-reported? If so, please make this clear and comment on whether the self-reporting of diagnoses is reliable and valid, if this is the case.

Page 7, line 169. Is it possible to include the survey questions in a supplementary file or include a link to the survey if available on a website? It would be useful to see the response options for each question.

Line 169-171. Please provide references for this sentence referring to research on potential relationships between factors and physical activity.

Lines 179-196. Please include whether these questionnaires included in the P10 were reliable and valid in the population you were looking at (high risk of CVD event, CVD diagnoses) or the general population.

Page 8, lines 199-212. Do any of these physical activity questions ask about intensity ie: MVPA? If not, please make this clear.

Page 10, line 254. Please remove bold letters for ‘MASS’.

Results

Page 11, line 284. Are these characteristics for included participants? If so, please make this clear.

Lines 28-287. Please include the total n for this subgroup in this sentence and in Figure 1.

Lines 287-289. Please include the total n for this subgroup in this sentence and in Figure 1.

Table 1. This table is very long. I suggest for dichotomous outcomes, only report one outcome. For other outcomes, possibly only report the most prevalent outcome.

In table 1, the past physical activity reported is much lower than the current physical activity, assuming both are reported a hours per week?

Page 16, lines 299-301. Please include the direction of these variable eg: higher past physical activity, if retired, adherence to the Mediterranean diet, and ? increased air pollution.

Line 301-304. The same applies to this sentence. Sometimes the direction is included but not consistently ie: is it a higher number of people living in the household?

Table 2. Please include n (%) in the third column to make this easier to interpret for the reader.

Table 3. Why is the p-value for BMI and environmental noise in bold as these p-values at not significant? Please also write p<0.001 in the last line of the table instead of including the exponential value.

Discussion

Page 28, Lines 369-380. Please highlight the gender findings here for gardening etc and total physical activity as you include this as a sub-heading later on.

Lines 382-388. I think this paragraph would fit better under the ‘Retirement’ sub-heading.

Page 29, line 411. I suggest ‘Gender’ should be the fourth heading as the first three factors were more prominent in your findings.

Lines 415-417. In the scoping review men were more physically active than women but did this also apply for gardening, etc and total physical activity or only for walking?

Page 31, lines 459-466. Also consider that assessment of physical activity should be broad, including activities such as gardening and household chores/work and total physical activity, particularly for females as your analysis shows that increased activity in these areas is associated with being female.

Page 32, lines 482-483. Please include in the Methods that the EPIC physical activity questionnaire has been validated.

Lines 488-489. Can you please add the sample size calculation for this analyses to the Methods?

Page 33, line 506. I think comorbidity and depression were both inversely associated with physical activity behavior. If this is correct, please include depression in this sentence.

Page 39. Reference 55 is the same as referenced 6. Please remove this duplicate.

Figure 1. Is it possible to improve the quality of this image as it is quite blurry?

Reviewer #2: Thank you for the opportunity to review this manuscript by Kulnik et al. The study addresses an important topic “Correlates of physical activity behaviour in a population sample with increased cardiovascular risk and established cardiovascular disease: data from the Paracelsus 10,000 prospective cohort study in Salzburg, Austria”. The use of data from the Paracelsus 10,000 study is a strength, and the topic is relevant for cardiovascular prevention. However, there are several concerns about the study design, statistical analysis, and interpretation require clarification before the findings can be appropriately interpreted.

Major comments

1) Methods: The manuscript repeatedly refers to the Paracelsus 10,000 study as a prospective cohort, which is accurate for the parent study. However, the present analysis appears to be a cross-sectional analysis of data from this cohort. This should be stated more explicitly throughout the manuscript, including in the title, abstract, methods, and discussion. I suggest revising the title to indicate that this is a “cross-sectional analysis of data from the Paracelsus 10,000 prospective cohort study” or similar. This clarification is important because the current wording may give readers the impression that the analysis itself is longitudinal or prospective.

2) Methods: The manuscript combines bivariate analyses, multivariable regression models, and machine-learning approaches. This is acceptable in principle, but these methods answer different questions and should be interpreted separately. Bivariate analyses describe crude associations, regression models estimate adjusted associations, and machine-learning models assess prediction or feature importance. These approaches should not be presented as equivalent forms of evidence for the same conclusion.

3) Methods: The use of bidirectional stepwise regression based on AIC also requires clearer justification. Stepwise procedures can produce unstable models, particularly when candidate predictors are correlated. If the aim is explanatory, a prespecified model based on prior evidence or a conceptual framework would be preferable. If the aim is prediction or variable selection, penalized regression approaches such as LASSO, ridge regression, or elastic net could be considered as alternatives or sensitivity analyses. LASSO can support sparse variable selection, ridge regression can help address multicollinearity, and elastic net may be useful when groups of predictors are correlated. These models could be tuned using cross-validation and compared with the reported machine-learning models.

4) Methods: The distinction between “regression” and “machine learning” should also be clarified, since regression models can also be considered supervised statistical-learning models. The more relevant distinction is between interpretable regression models for adjusted associations and more flexible prediction-oriented models such as random forests or gradient boosting.

5) Methods: The manuscript includes sociodemographic, clinical, psychological/cognitive, lifestyle, and environmental variables as candidate correlates of physical activity behaviour. However, these variable groups differ substantially in their temporal and causal interpretation. Clinical variables such as CVD risk, comorbidity, and body mass index may be causes of lower physical activity, consequences of prior physical activity, mediators, or bidirectionally related to physical activity. This issue is particularly important in a cross-sectional analysis. For example, higher BMI or musculoskeletal pain may reduce current physical activity, but long-term low physical activity may also contribute to these clinical conditions. The authors should therefore avoid presenting these variables as determinants or intervention targets unless temporal ordering can be supported. A clearer conceptual framework, such as a table or directed acyclic graph classifying variables as potential confounders, exposures, mediators, or consequences, would improve interpretation.

The role of “CVD risk” also requires clarification. It appears to be used both as part of the sample definition or subgroup classification and as a clinical correlate. The authors should specify exactly how this variable was included in the regression and machine-learning models, and whether it represents SCORE2/SCORE2-OP category, established CVD status, or the combined primary/secondary prevention grouping. Possible overlap with variables such as age, sex, smoking, BMI, and comorbidity should also be addressed.

6) Results: Because the analysis is cross-sectional, the results should not be interpreted as evidence that the identified variables cause or determine physical activity behaviour. Terms such as “predict,” “influence,” and implications for personalised physical-activity promotion should be used cautiously unless the authors clearly mean statistical prediction rather than temporal or causal prediction.

7) The Results section should more clearly distinguish findings from bivariate analyses, multivariable regression models, and machine-learning analyses. Please specify which findings apply to the primary outcome and which apply to secondary outcomes, and indicate whether each finding is model-specific or consistent across analytical approaches. Also, effect estimates should be reported and interpreted more consistently. The relatively low explained variance of the primary regression model should also be acknowledged, as it indicates that the identified variables explain only a limited proportion of variability in physical activity behaviour.

8) Results: For the machine-learning analyses, SHAP values should be interpreted as contributions to model prediction, not as evidence of causal or clinical importance. If model performance was similar across algorithms, conclusions based on the single best-performing model should be presented cautiously.

9) The Discussion should more clearly state that the findings represent cross-sectional associations, not determinants of physical activity behaviour. Statements about prevention, tailoring, or personalised physical-activity promotion should be softened unless supported by longitudinal or interventional evidence. Finally, the possibility of reverse causation should be discussed, particularly for clinical variables such as BMI, pain, comorbidity, and depression, which may both influence and be influenced by physical activity.

Minor comments

10) The Abstract should be revised for clarity and precision. I think, the total size of the original Paracelsus 10,000 cohort does not need to be reported in the Abstract; the focus should be on the analytical sample used in the present study. I suggest moving the analytical sample size, mean age, and female proportion to the first sentence of the Results section.

The phrase “greater (co)morbidity” is vague and should be specified. Please indicate which comorbidity variable or specific comorbidities were associated with physical activity behaviour.

The Methods section of the Abstract states that stepwise multiple regression and machine-learning models were used, but the specific machine-learning model(s) should be briefly indicated. The Results section should also clarify which model produced the reported effect estimates and what metric these estimates represent, for example adjusted regression coefficients for weekly hours of walking, cycling, and sports. At present, the Abstract results are somewhat difficult to interpret.

11) Methods: the bivariate analysis section should explain how the authors chose between Pearson’s and Spearman’s correlation coefficients. Please specify whether Pearson’s correlation was used for approximately linear relationships between continuous variables, and whether Spearman’s correlation was used for ordinal variables, skewed distributions, outliers, or monotonic but non-linear relationships. Or how the normality of continuous variables were assessed.

12) Discussion-Limitation: The large reduction from the eligible cohort to the complete-case analytical sample should be more clearly acknowledged as a potential source of selection bias. If participants with complete data differed from excluded participants in characteristics related to physical activity, the reported associations may not be fully generalizable to the broader population with increased cardiovascular risk or established cardiovascular disease.

**********

what does this mean?). If published, this will include your full peer review and any attached files.

If you choose “no”, your identity will remain anonymous but your review may still be made public.

**Do you want your identity to be public for this peer review?**  For information about this choice, including consent withdrawal, please see our Privacy Policy

Reviewer #1: No

Reviewer #2: **Yes:** Sina Kazemian

**********

[NOTE: If reviewer comments were submitted as an attachment file, they will be attached to this email and accessible via the submission site. Please log into your account, locate the manuscript record, and check for the action link "View Attachments". If this link does not appear, there are no attachment files.]

To ensure your figures meet our technical requirements, please review our figure guidelines: https://journals.plos.org/plosone/s/figures

You may also use PLOS’s free figure tool, NAAS, to help you prepare publication quality figures: https://journals.plos.org/plosone/s/figures#loc-tools-for-figure-preparation.

NAAS will assess whether your figures meet our technical requirements by comparing each figure against our figure specifications.

# Author comment: Author response to Decision Letter 1

**DOI:** 10.1371/journal.pone.0356607.r002

23 Jul 2026

Please refer to the attached file "Response to Reviewers".

Attachment Submitted filename: Response to Reviewers.docx

# Peer review: Decision Letter 1

**DOI:** 10.1371/journal.pone.0356607.r003

**Authors:** Seyed Aria Nejadghaderi

5 Aug 2026

Correlates of physical activity behaviour in a population sample with increased cardiovascular risk and established cardiovascular disease: cross-sectional analysis of data from the Paracelsus 10,000 prospective cohort study in Salzburg, Austria

PONE-D-26-13124R1

Dear Dr. Kulnik,

We’re pleased to inform you that your manuscript has been judged scientifically suitable for publication and will be formally accepted for publication once it meets all outstanding technical requirements.

Within one week, you’ll receive an e-mail detailing the required amendments. When these have been addressed, you’ll receive a formal acceptance letter and your manuscript will be scheduled for publication.

An invoice will be generated when your article is formally accepted. Please note, if your institution has a publishing partnership with PLOS and your article meets the relevant criteria, all or part of your publication costs will be covered. Please make sure your user information is up-to-date by logging into Editorial Manager at Editorial Manager® and clicking the ‘Update My Information' link at the top of the page. For questions related to billing, please contact billing support.

If your institution or institutions have a press office, please notify them about your upcoming paper to help maximize its impact. If they’ll be preparing press materials, please inform our press team as soon as possible -- no later than 48 hours after receiving the formal acceptance. Your manuscript will remain under strict press embargo until 2 pm Eastern Time on the date of publication. For more information, please contact onepress@plos.org.

Kind regards,

Seyed Aria Nejadghaderi

Academic Editor

PLOS One

Additional Editor Comments (optional):

Reviewers' comments:

Reviewer's Responses to Questions

**Comments to the Author**

Reviewer #1: All comments have been addressed

**********

2. Is the manuscript technically sound, and do the data support the conclusions?

Reviewer #1: Yes

**********

3. Has the statistical analysis been performed appropriately and rigorously?

Reviewer #1: Yes

**********

4. Have the authors made all data underlying the findings in their manuscript fully available?

The PLOS Data policy

Reviewer #1: Yes

**********

5. Is the manuscript presented in an intelligible fashion and written in standard English?

Reviewer #1: Yes

**********

Reviewer #1: Thank you for thoroughly addressing my comments and the comments of the other reviewer, which I believe has improved the manuscript.

**********

what does this mean?). If published, this will include your full peer review and any attached files.

If you choose “no”, your identity will remain anonymous but your review may still be made public.

**Do you want your identity to be public for this peer review?**  For information about this choice, including consent withdrawal, please see our Privacy Policy

Reviewer #1: No

**********

# Editor report: Acceptance letter

**DOI:** 10.1371/journal.pone.0356607.r004

**Authors:** Seyed Aria Nejadghaderi

PONE-D-26-13124R1

PLOS One

Dear Dr. Kulnik,

I'm pleased to inform you that your manuscript has been deemed suitable for publication in PLOS One. Congratulations! Your manuscript is now being handed over to our production team.

At this stage, our production department will prepare your paper for publication. This includes ensuring the following:

* All references, tables, and figures are properly cited

* All relevant supporting information is included in the manuscript submission,

* There are no issues that prevent the paper from being properly typeset

You will receive further instructions from the production team, including instructions on how to review your proof when it is ready. Please keep in mind that we are working through a large volume of accepted articles, so please give us a few days to review your paper and let you know the next and final steps.

Lastly, if your institution or institutions have a press office, please let them know about your upcoming paper now to help maximize its impact. If they'll be preparing press materials, please inform our press team within the next 48 hours. Your manuscript will remain under strict press embargo until 2 pm Eastern Time on the date of publication. For more information, please contact onepress@plos.org.

You will receive an invoice from PLOS for your publication fee after your manuscript has reached the completed accept phase. If you receive an email requesting payment before acceptance or for any other service, this may be a phishing scheme. Learn how to identify phishing emails and protect your accounts at https://explore.plos.org/phishing.

If we can help with anything else, please email us at customercare@plos.org.

Thank you for submitting your work to PLOS One and supporting open access.

Kind regards,

PLOS ONE Editorial Office Staff

on behalf of

Dr. Seyed Aria Nejadghaderi

Academic Editor

PLOS One
